# A Stage-Specific OTX2 Regulatory Network and Maturation-Associated Gene Programs Are Inherent Barriers to RPE Neural Competency

**DOI:** 10.3389/fcell.2022.875155

**Published:** 2022-04-19

**Authors:** Jared A. Tangeman, J. Raúl Pérez-Estrada, Emily Van Zeeland, Lin Liu, Alexandra Danciutiu, Erika Grajales-Esquivel, Byran Smucker, Chun Liang, Katia Del Rio-Tsonis

**Affiliations:** ^1^ Department of Biology and Center for Visual Sciences, Miami University, Oxford, OH, United States; ^2^ Department of Statistics, Miami University, Oxford, OH, United States; ^3^ Department of Computer Science and Software Engineering, Miami University, Oxford, OH, United States

**Keywords:** RPE, retina, ATAC-seq, reprogramming, regeneration, Otx2

## Abstract

The retinal pigment epithelium (RPE) exhibits a diverse range of plasticity across vertebrates and is a potential source of cells for the regeneration of retinal neurons. Embryonic amniotes possess a transitory ability to regenerate neural retina through the reprogramming of RPE cells in an FGF-dependent manner. Chicken RPE can regenerate neural retina at embryonic day 4 (E4), but RPE neural competence is lost by embryonic day 5 (E5). To identify mechanisms that underlie loss of regenerative competence, we performed RNA and ATAC sequencing using E4 and E5 chicken RPE, as well as at both stages following retinectomy and FGF2 treatment. We find that genes associated with neural retina fate remain FGF2-inducible in the non-regenerative E5 RPE. Coinciding with fate restriction, RPE cells stably exit the cell cycle and dampen the expression of cell cycle progression genes normally expressed during regeneration, including *E2F1*. E5 RPE exhibits progressive activation of gene pathways associated with mature function independently of retinectomy or FGF2 treatment, including retinal metabolism, pigmentation synthesis, and ion transport. Moreover, the E5 RPE fails to efficiently repress *OTX2* expression in response to FGF2. Predicted OTX2 binding motifs undergo robust accessibility increases in E5 RPE, many of which coincide with putative regulatory elements for genes known to facilitate RPE differentiation and maturation. Together, these results uncover widespread alterations in gene regulation that culminate in the loss of RPE neural competence and implicate OTX2 as a key determinant in solidifying the RPE fate. These results yield valuable insight to the basis of RPE lineage restriction during early development and will be of importance in understanding the varying capacities for RPE-derived retinal regeneration observed among vertebrates.

## Introduction

A longstanding goal for vision restoration therapies is to develop methods that can employ endogenous cell sources to replace retinal neurons. Various cell types have been explored as potentially viable candidates for generating retinal neurons, and in particular, a great amount of attention has been given to the cells of the retinal pigment epithelium (RPE) and Müller glia (Chiba, 2014; Lahne et al., 2020). RPE cells represent a promising avenue for exploration, as this cell population exhibits a diverse range of plasticity across vertebrate contexts. For example, RPE cells can regenerate retinal neurons in embryonic amniotes and certain urodele amphibians (Tsonis and Del Rio-Tsonis, 2004). In contrast, following retinal injury, mammalian RPE is limited to non-regenerative wound closure, which can be observed in fibrotic pathologies such as proliferative vitreoretinopathy (Chiba, 2014). Importantly, both the RPE and neural retina are indispensable for vision, and throughout an organism’s lifetime, these two layers will maintain an intimate functional relationship. The RPE is a monolayer of polygonal, pigmented cells adjacent to the outer layer of the neural retina. RPE cells directly contact photoreceptor outer segments and phagocytize shed photoreceptor discs (Yang et al., 2021). Importantly, RPE cells constitute a major component of the blood-retinal barrier and are necessary for photoreceptor maintenance, retinal cycling, and proper light absorption (Yang et al., 2021). In order to assess the viability of RPE cells as a source of retinal neurons, it is imperative to better understand the gene regulatory mechanisms that establish and maintain RPE cell identity, and how these mechanisms may impede neural competence in non-regenerative contexts.

Both the RPE and neural retina are embryonically derived from the optic vesicle, a rudimentary structure of the developing eye. The optic vesicle is a bilayer structure comprised of multipotent progenitor cells, and these cells differentiate under the direction of secreted patterning factors, such as FGF, TGF-β, and WNT family proteins (Bharti et al., 2006; Fuhrmann, 2010; Fuhrmann et al., 2014). These factors act as environmental cues that establish positional identity throughout the optic vesicle, and ultimately the outer cell layer adopts an RPE fate, while the inner optic vesicle will form a neuroepithelium that gives rise to a laminar neural retina. During retinal differentiation, FGF signals are secreted from the anterior surface ectoderm and bias progenitor cells toward the neural retina lineage (Guillemot and Cepko, 1992; Pittack et al., 1997). FGF signaling restricts the transcription factor MITF to the RPE layer, while the inner neuroepithelium becomes demarcated by the transcription factor VSX2 (Liu et al., 1994; Nguyen and Arnheiter, 2000). Although MITF and VSX2 are two of the earliest indicators of optic vesicle regionalization, they are part of an intricate gene regulatory network that is not fully defined. A recent study leveraged gene expression and chromatin accessibility profiling to resolve intrinsic transcription factor networks that delineate early RPE and neural retina cell differentiation (Buono et al., 2021). This study uncovered a conserved, sequential transcription factor program that specifies the RPE and activates functional attributes, exemplified by the TEAD-mediated activation of RPE desmosome function.

Following the onset of differentiation, RPE retains multipotent qualities for a short period of time. During this time, the ectopic placement of FGFs within the presumptive RPE layer can induce RPE cells to reprogram and adopt a neural retina identity (Zhao et al., 2001). FGF-induced RPE reprogramming has been demonstrated across numerous amniotic models, including the embryonic mouse, rat, and chicken (Park and Hollenberg, 1989; Zhao et al., 1995, 2001; Spence et al., 2004). However, RPE fate restriction occurs rapidly during differentiation, after which RPE cells become refractory to FGF and ostensibly lose their neural competence. Chicken RPE can be reprogrammed to neural retina at embryonic day 4 (E4), but neural competence is abrogated by embryonic day 5 (E5) (Pittack et al., 1997; Sakami et al., 2008). The mechanisms that lead to the loss of RPE neural competency are not fully resolved, but TGF-β signals secreted from the underlying periocular mesenchyme are known to promote the RPE fate (Fuhrmann et al., 2000). Treatment of chicken RPE explants with TGF-β signaling inhibitors is sufficient to prolong the window during which RPE can undergo FGF-inducible neural reprogramming (Sakami et al., 2008), but it is unclear how TGF-β signals intersect with FGF cues to establish cell fate.

Recent studies have characterized the molecular events that allow E4 RPE cells to shift their fate in response to FGF2. RPE reprogramming in the embryonic chicken proceeds through the sequential steps of RPE dedifferentiation, the proliferation of a transitory population of transit-amplifying cells, and finally differentiation of progenitors toward neural lineages (Luz-Madrigal et al., 2014). This process can be readily induced *via* surgical removal of the retina (retinectomy) followed by the delivery of an FGF2-containing bead to the eye cup. Reprogrammed RPE forms a neuroepithelium of retinal progenitor cells within 3 days post-retinectomy (PR), and within 7 days, these progenitors will differentiate to produce all major retinal cell types (Spence et al., 2004). Importantly, the incipient transcriptional features of reprogramming are detectable in the RPE as early as 6 h post-retinectomy (6hPR), although the cells remain non-proliferative during this acute window (Luz-Madrigal et al., 2014; Tangeman et al., 2021). During this time, RPE cells increase expression of genes associated with the neural retina fate, such as *VSX2* and *ASCL1*, as well as genes encoding eye field transcription factors, such as *SIX6*, *RAX*, and *LHX2*. Simultaneously, the reprogramming RPE cells begin to shed their RPE identity, characterized by loss of *MITF* and *TYR* expression. These early transcriptional events are coordinated with extensive epigenomic rearrangements, including global reductions in DNA methylation and repressive histone marks (Luz-Madrigal et al., 2020). Although RPE reprogramming is an FGF-dependent process, some of the transcriptional and epigenetic signatures of RPE reprogramming are inducible by retinectomy alone (Luz-Madrigal et al., 2014; Tangeman et al., 2021). Despite these defined mechanisms, it is unclear how changes in gene regulation lead to a loss of neural competency at later stages of RPE differentiation.

In order to identify specific mechanisms that underlie the loss of RPE neural competence, we directly compare retinectomy and FGF2 treatment responses in the chicken RPE at plastic (E4) and fate restricted (E5) stages of development. Using gene expression and chromatin accessibility profiling, we uncover neural gene signatures that remain FGF2-inducible past the window of lineage restriction, such as *VSX2* and *ASCL1* expression. Moreover, we determine that RPE neural competency strictly correlates with the basal proliferative status of RPE during development. Coinciding with fate restriction, the RPE undergoes widespread activation of gene programs associated with mature RPE function, such as pigmentation, retinal metabolism, and ion transport, and these gene sets remain stably expressed independently of retinectomy or FGF2 treatment. Many of these gene signatures are associated with stage-specific changes in chromatin accessibility, and we identify the global dysregulation of accessibility at a diverse set of transcription factor binding sites. Finally, we observe that the E5 RPE fails to efficiently silence the RPE specifying factor *OTX2* in response to FGF2, and that the accessibility of putative OTX2 binding motifs are greatly enhanced at this stage. Collectively, these results uncover extensive shifts in gene regulation that correlate with the loss of RPE neural competence and implicate OTX2 as a primary determinant in solidifying the RPE fate.

## Materials and Methods

### Chick Embryos and Retinectomy

Fertile SPAFAS pathogen-free chicken (*Gallus gallus*) eggs from Charles River Laboratories (Charles River Laboratories, cat. 10100329) were used for ATAC-seq and RNA-seq experiments. For all other experiments, fertile White Leghorn chicken eggs obtained from Michigan State University were used. Eggs were incubated in a humidified, rotating incubator at 38°C before performing experiments at Hamburger Hamilton stage 24 (E4) or 26 (E5). Retinectomy was performed as previously described (Spence et al., 2004). For FGF2 delivery, acrylic beads with immobilized surface heparin (Millipore Sigma, cat. H5263) were soaked overnight at 4°C in bovine basic FGF2 (R&D Biosystems, cat. 133-FB-025) at a ratio of 20 beads per 1 µg of FGF2 in 4 µl of sterile PBS. Two to three beads per eye cup were delivered at the time of surgery.

### RNA-Seq Library Preparation and Sequencing

Embryos were collected into a petri dish containing cold, sterile HBSS without Mg^2+^ or Ca^2+^ and a single eye from each embryo was enucleated and washed. The anterior chambers of eyes were discarded, and the neural retina was peeled away before gently debriding mesenchyme from the basal surface of the RPE. Three isolated sheets of RPE were collected per biological sample and collected directly into 1X DNA/RNA Shield (Zymo, cat. R1200) on ice. RNA was isolated using the Quick-RNA Miniprep Kit (Zymo, cat. R1054) per manufacturer’s instructions, including 15 minutes of in-column DNase I treatment. RNA integrity was assayed with the Bioanalyzer RNA 6000 Pico Kit (Agilent, cat. 5067-1513) and samples with RIN value >9 were used for sequencing. RNA libraries were constructed at the DNA Link, Inc. sequencing core (Los Angeles, CA, United States) using the TruSeq Stranded mRNA Library Prep kit (Illumina, cat. 20020594) with 580 ng–1 µg of total RNA per sample. Libraries were sequenced on the Illumina NovaSeq 6000 with 100 base pair paired end reads to a depth of >30 million read pairs per sample.

### RNA-Seq Data Analysis

Raw reads were quality analyzed using FastQC and MultiQC (Ewels et al., 2016; Babraham Bioinformatics - FastQC, 2020). Low-quality ends and adapters were trimmed using Cutadapt and Trim Galore with the parameters *--stringency 3 --length 36* (Martin, 2011; Krueger, 2016). Chicken genome build GRCg6a was indexed using the STAR aligner, incorporating splice junctions from the Ensembl GTF annotation file and the parameter *--sjdbOverhang 99* (Dobin et al., 2013; Howe et al., 2021). Two-pass alignment with STAR was employed and the parameters *--quantMode GeneCounts TranscriptomeSAM --readFilesCommand zcat --outSAMstrandField intronMotif --outSAMtype BAM SortedByCoordinate* were used. Gene counts were generated using Stringtie and Ensembl chicken gene annotation release 101 (Pertea et al., 2015; Howe et al., 2021). Differential expression testing was performed with DESeq2, and genes with less than 10 raw counts detected across all conditions were dropped prior to analysis (Love et al., 2014). Clustering of DEGs was performed with affinity propagation clustering (Frey and Dueck, 2007). Row-scaling of expression values was performed by applying log_10_(normalized count + 0.5) to all expression values. Subsequently, mean gene expression across all conditions was subtracted from each value and divided by standard deviation. Pathway enrichment analysis was performed with the Metascape online tool using human analysis (Zhou et al., 2019). Gene set enrichment analysis (GSEA) was performed on the normalized count matrix obtained from DESeq2 and chicken genes were converted to human orthologs (Mootha et al., 2003; Subramanian et al., 2005). GSEA was performed using 1000 permutations and gene set permutations with gene set size filters min = 15 and max = 500. All figures were generated in the R environment. For the RNA-seq genome browser, tracks were normalized to Transcripts Per Million (TPM) by converting bam files to bigwig format with the bamCoverage tool and parameters *--effectiveGenomeSize 1065365434* and *--normalizeUsing BPM* and visualized using the Integrative Genomics Viewer (Thorvaldsdóttir et al., 2013; Ramírez et al., 2016). Normalized replicates were collapsed prior to visualization using bigWigMerge (Kent et al., 2010). Differentially expressed genes (DEGs) are defined throughout by an adj. *p*-value ≤ 0.05 and no log fold change (LFC) criteria are applied unless otherwise stated.

### ATAC-Seq Library Preparation and Sequencing

ATAC-seq library preparation was performed using ATAC-Seq Kit (Active Motif cat. 53150) per manufacturer’s instructions. Briefly, RPE was isolated in cold PBS as described above for RNA collection. Two isolated sheets of RPE were collected per biological sample and placed on ice. RPE cells were lysed in cold ATAC-Seq Kit lysis buffer and nuclei were counted with a hemocytometer. Approximately 100,000 RPE nuclei from two embryos were aliquoted for Tn5 tagmentation per sample. Final libraries were quality validated using the Bioanalyzer HS DNA Kit (Agilent, cat. 5067-4626) and quantified using the Qubit 4 Fluorometer with the dsDNA Quantitation, high sensitivity kit (ThermoFisher Scientific, cat. Q32851). Validated libraries were sequenced at the Novogene sequencing core (Sacramento, CA, United States) on an Illumina HiSeq Series sequencing platform to a minimum depth of 61 million read pairs per sample using 150 base pair reads.

### ATAC-Seq Data Analysis

Reads were trimmed using cut adapt and trim galore with the trimming parameters *--clip_R1 16 --clip_R2 18 --three_prime_clip_R1 6 --three_prime_clip_R2 4*. Alignment of raw reads to chicken genome GRCg6a was performed with Bowtie2 (Langmead and Salzberg, 2012) using the parameters *--very-sensitive -k 5 -p 40*. Aligned reads were deduplicated using Picard tools (Picard Toolkit, 2019). Peaks were called for samples individually with HMMRATAC (Tarbell and Liu, 2019). HMMRATAC defined open regions were unified across all samples into a consensus peak file using BEDtools *merge -d 10 -I* (Quinlan and Hall, 2010). Peaks aligning to the mitochondrial chromosome were discarded, and peak counts were determined using featureCounts with parameters *-F SAF -s 0 -T 10* (Liao et al., 2014). Conversely, peak summits were defined by the HMMRATAC summit regions, which were extended by 50 bps and collapsed into a consensus summit file as defined above. Differential expression testing of peak regions and peak summits was performed with DESeq2 (Love et al., 2014). ATAC coverage tracks were visualized using the Integrative Genomics Viewer (Thorvaldsdóttir et al., 2013). Prior to visualization, bam files were converted to bigwig format and normalized using the bamCoverage *--binSize 1* and *--scaleFactor* parameter using the inverse of DESeq2 size factors (Ramírez et al., 2016). For combined visualization tracks, normalized replicates were collapsed using bigWigMerge (Kent et al., 2010). Accessibility heatmaps were generated using deepTools computeMatrix reference-point and plotHeatmap functions (Ramírez et al., 2016). Motif detection and peak annotation to the nearest transcript start site (TSS) was performed using the HOMER software (Heinz et al., 2010). Assignment of peaks to genomic features was performed using ChIPseeker (Yu et al., 2015). HOMER motif analysis was conducted with the script findMotifsGenome.pl and parameter *-size given* with the standard HOMER motif library. Binned motif analysis and k-mer analysis was executed using the monaLisa R package (Machlab et al., 2022) and the JASPAR 2022 vertebrate motif database (Castro-Mondragon et al., 2022). For monaLisa analysis, all differentially accessible peak summits (|LFC| ≥ 1 and adj. *p*-value ≤ 0.05) were segmented into seven bins by LFC across the E4 and E5 conditions. A cut-off of −log(adj. *p*-value) > 3 in any non-0 bin was applied to all monaLisa motif and k-mer results. The annotation of peak summits for the presence of transcription factor binding summits was performed using the OTX2 position weight matrix and monaLisa function findMotifHits.

### Histology and EdU Fluorescence Detection

Hematoxylin and eosin staining was performed as previously described (Luz-Madrigal et al., 2014). EdU proliferation assay was conducted with Click-iT EdU Cell Proliferation Kit (ThermoFisher Scientific, cat. C10337) per manufacturer’s instructions. Briefly, 30 µl of 3.3 mM EdU in sterile PBS was added to embryos 1 h before collection. Embryos were washed in PBS and fixed overnight in 4% paraformaldehyde at 4°C. Embryos were then equilibrated in 30% sucrose solution overnight and snap frozen in a bath of dry ice and ethanol in OCT media. Cryo-sections were permeabilized with 1% Saponin for 5 minutes before the Click-iT reaction, and DAPI (Millipore Sigma, cat. 10236276001) staining was performed immediately before applying Fluoromount Aqueous Mounting Medium (Sigma, cat. F4680-25 ML) and coverslip. Fluorescence imaging was performed using a Zeiss 710 Laser Scanning Confocal System with sequential imaging of channels and pinhole set to 1.0 airy unit. For quantification of proliferating cells, EdU+ cells were counted as a proportion of total RPE nuclei. Due to possible differences in the variation among the groups, statistical inference for EdU assays was performed using the Kruskal-Wallis nonparametric test followed by pairwise Dunn’s test of all treatment pairs with a Benjamini and Yekutieli *p*-value multiplicity adjustment (Dunn, 1964; Benjamini and Yekutieli, 2001). Statistics were performed in the R environment using rstatix version 0.7.0 (Kassambara, 2021).

### RPE Explants

RPE explant culture was performed as previously described (Sakami et al., 2008), with the following modifications. Briefly, RPE and a small amount of underlying mesenchyme were dissected as posterior eye cup explants from chicken embryos at Hamburger and Hamilton stage 24 (E4) and 26 (E5) in modified HBSS (without calcium chloride, magnesium sulfate, and sodium bicarbonate) supplemented with 5 mM HEPES and 0.6% D-glucose. The explants were washed three times in HBSS solution and cultured in 500 µl of culture medium (DMEM/F12, supplemented with 0.9% D-glucose, 0.1125% NaHCO3, 20 mM HEPES, 5% FBS, 100 U/ml of penicillin, and 100 μg/ml of streptomycin) in 24-well culture plates. The plates were incubated in an orbital shaker at 50 rpm (3-D, Fixed Tilt Platform Rotator, Grant Instruments), 37°C, and 5% CO_2_. FGF2 (R&D Systems, cat. 3718-FB-025) was added at a concentration of 100 ng/µl as specified.

### RT-qPCR Gene Expression and Statistics

RPE explants were collected in 200 µl of DNA/RNA shield buffer (Zymo, cat. 1220-25) and stored at −20°C. Total RNA was isolated using Quick-RNA Miniprep Plus Kit Microprep (Zymo, cat. R1057), following manufacturer’s instructions. Total RNA was analyzed for quantity and quality using Nanodrop ND-2000 Spectrophotometer (Thermo Scientific) and the Agilent 2100 Bioanalyzer (Agilent Technologies), respectively. 200 ng of RNA was used as a template to synthesize cDNA using QuantiTect Reverse Transcription kit (Qiagen, cat. 205313) according to manufacturer’s instructions. The synthesized cDNA was diluted at 1:10 ratio and 2 µl of this dilution were used for quantitative PCR (qPCR) reaction. The final reaction mix contained: 2 µl of diluted cDNA, 5 µl of RT2 SYBR Green Master Mix (Qiagen, cat. 204074) and 50 nM of each primer, adjusted to 20 µl with water. qPCR reactions were set up in duplicate in the Rotor-Gene Q thermocycler 5 plex (Qiagen). Primers reported here were designed using primer blast (Ye et al., 2012) and obtained from IDT Technologies (Table 1). The comparative ΔΔCt method was used to determine relative gene expression levels compared to a housekeeping gene (RPLP0) and relative mRNA was normalized to explants immediately collected after dissection (time 0). Each biological sample contained three pooled explants. Four biological samples per time point and condition were used. We first analyzed the data using ANOVA with Dunnett’s adjustment for multiple comparisons with the control (time 0) condition (Dunnett, 1955), which provided strong evidence of differences between treatments and control for at least one time point in all qPCR datasets. However, this procedure requires an assumption that the responses are normally distributed and have the same variance within each group. Subsequently, we applied Levene’s test for homoscedasticity (Fox and Weisberg, 2019), and residual values were plotted for subjective assessment of distribution and variance. The normality and equal variance assumptions are questionable for at least some groups associated with three out of the 18 gene sets analyzed. Thus, we reanalyzed these data using a nonparametric Kruskal-Wallis test followed by Dunn’s procedure with Benjamini-Yekutieli adjustment for multiple comparisons with a control (Dunn, 1964; Benjamini and Yekutieli, 2001). This is a much more conservative test and given the small sample sizes (*n* = 4) is only approximate, but it relaxes the normality and equal variance assumptions and still provides evidence of differences with control for the three genes in question at several time points (Supplementary File S1). All significance values shown in the RT-qPCR figures are derived from Dunnett’s Test, and Dunn’s Test *p*-values are also explicitly stated in the text where parametric assumptions are in question. In the interest of transparency, in the process of analyzing these data, we also used a version of Dunn’s procedure that compared all pairs of means, rather than just comparing treatments to control. This was even more conservative, and because it includes comparisons that don’t include control, it is less appropriate than the procedure we used, and thus we discarded the results. All statistics described for RT-qPCR were performed in the R environment using rstatix version 0.70 (Kassambara, 2021), car version 3.0-12 (Fox and Weisberg, 2019), DescTools version 0.99.44 (Signorell et al., 2021), PMCMRplus version 1.9.3 (Pohlert, 2021), and stats version 4.1.2 (R Core Team, 2021).

**TABLE 1:** List of primers used in qPCR.

Gene	GenBank ID	Forward primer	Reverse primer
*ASCL1*	NM_204412.1	5ʹ-CCC​GGC​AGG​ACG​CTC-3ʹ	5ʹ-GGG​GAG​AGG​AAA​ACG​CAA​CA-3ʹ
*E2F1*	NM_205219.1	5ʹ-GGA​CGA​TCT​CAT​CCA​GAC​GTG-3ʹ	5ʹ-ACG​TAG​GCT​GCG​TGC​T-3ʹ
*MITF*	NM_205029.1	5ʹ-CCC​AAA​TCA​AAC​GAC​CCG​GAT​A-3ʹ	5ʹ-GTG​CGT​TGC​TGC​TCT​CTT​TG-3ʹ
*OTX2*	NM_204520.2	5ʹ-GTC​GGT​TAT​CCC​GCC​ACC-3ʹ	5ʹ-TTT​TCA​AGG​CCA​CCT​CCT​CC-3ʹ
*PAX6*	NM_205066.1	5ʹ-GGC​AGA​AGA​TCG​TGG​AAC​TC-3ʹ	5ʹ-TTC​GTA​ATA​CCT​GCC​CAA​AA-3ʹ
*RPE65*	NM_204884.1	5ʹ-CCT​ACC​ACC​GGA​GGT​TTG​TT-3ʹ	5ʹ-GGT​CTG​GGT​AGG​CGT​AGG​TA-3ʹ
*RPLP0*	NM_204987.2	5ʹ-GGA​GCT​CAC​AGC​TCG​TCT​TT-3ʹ	5ʹ-TAG​TTG​GAC​TTC​CAC​GTC​GC-3ʹ
*SIX6*	NM_001389365.1	5ʹ-AGG​TGG​GCA​ACT​GGT​TCA​AA-3ʹ	5ʹ-CTG​CTG​CTG​TAG​CCT​GTT​CT-3ʹ
*SOX2*	NM_205188.2	5ʹ-TGA​ACG​GAT​CGC​CTA​CCT​AC-3ʹ	5ʹ-CTG​GAT​TCC​GTC​TTG​ACC​AC-3ʹ
*TYR*	NM_204160.1	5ʹ-TTT​GCT​GAT​CCA​CAC​ACT​GC-3ʹ	5ʹ-GAT​CAT​TCG​CAG​AGC​CTT​GT-3ʹ
*VSX2*	NM_204768.1	5ʹ-CAG​ACG​GCC​AGC​TCA​GAT​TC-3ʹ	5ʹ-AGG​CCT​TTT​CCA​GCT​CTT​CC-3ʹ

## Results

### Alterations in Gene Regulation and Chromatin Accessibility Underlie RPE Differentiation State and Reprogramming Competency

**FIGURE 1:**
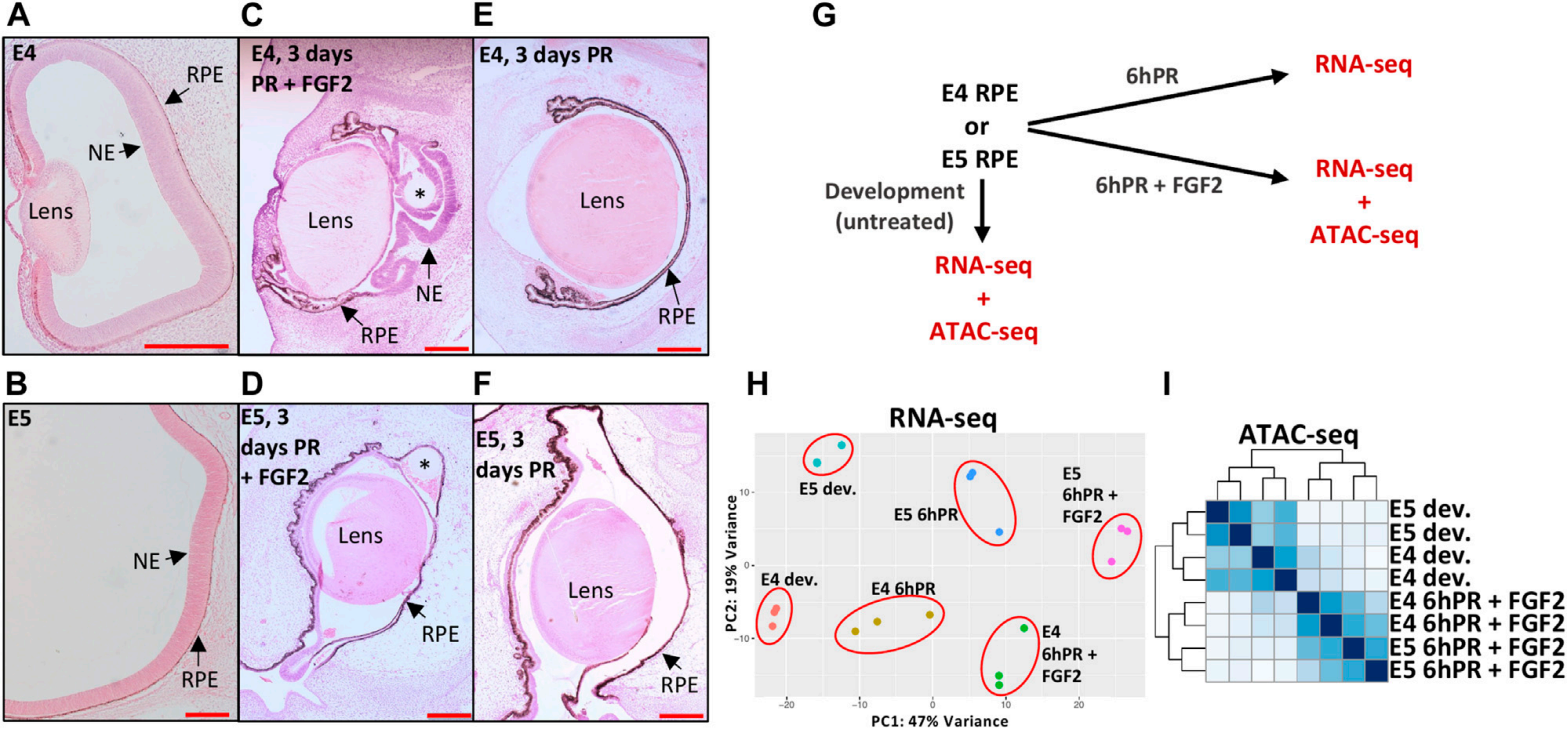
Profiling gene expression and genomic accessibility in the plastic and fate-restricted RPE. The developing (dev.) eye at E4 **(A)** and posterior eye at E5 **(B)** are shown with RPE and neuroepithelium (NE; presumptive neural retina) labeled. A regenerating NE is observed 3 days PR if treated with FGF2 at E4 **(C)**, but no regeneration is observed 3 days PR and FGF2 treatment at E5 **(D)**. At 3 days PR, no regeneration is observed if surgery is performed at E4 **(E)** or E5 **(F)** in the absence of FGF2. Scale bars in **(A**–**F)** are each 200 μm. Asterisk (*) denotes the FGF2 bead. **(G)** Schematic summarizes the collection of RNA-seq and ATAC-seq samples used in this study. **(H)** Principal component analysis summarizes the variation present in RNA-seq normalized gene expression values across 18 samples and six tested conditions. **(I)** Heatmap displays blind hierarchical clustering of normalized ATAC-seq peak signal for each sample.

In order to interrogate the gene regulatory underpinnings of RPE lineage commitment, we first sought to characterize changes in gene regulation that differentiate the plastic E4 RPE (Figure 1A) from the fate restricted E5 RPE (Figure 1B). Retinectomy and delivery of an FGF2-containing bead to the eye cup resulted in a robust regenerative response, in which we observed significant domains of RPE that had been reprogrammed to a neuroepithelium 3 days PR (Figure 1C), consistent with previous reports (Coulombre and Coulombre, 1965; Park and Hollenberg, 1989; Spence et al., 2004). In contrast, we did not observe any RPE reprogramming 3 days PR with FGF2 treatment when the surgery was performed at E5 (Figure 1D), which is consistent with reports that RPE cell fate becomes restricted by E5 of chicken development (Pittack et al., 1997; Sakami et al., 2008). Similarly, we did not observe RPE reprogramming at either stage if the retinectomy was performed in the absence of exogenous FGF2 (Figures 1E,F). After validating this phenotype *in vivo*, we next performed bulk mRNA sequencing (RNA-seq) on RPE isolated from chicken eyes at E4 and E5 from three conditions (Figure 1G): developing, 6hPR, and 6hPR with FGF2 treatment (6hPR + FGF2). Principal component analysis of RNA-seq derived expression values revealed a spatial separation of samples by retinectomy and FGF2 treatment along component 1 and by developmental stage along component 2, pointing toward retinectomy and FGF2 treatment as the primary sources of variation within the observed transcriptional states (Figure 1H). In addition, the RNA-seq data displayed high per-base quality, alignment rates to the chicken genome above 92%, uniform GC content, and all assayed samples clustered by treatment, pointing toward overall high quality gene expression data (Supplementary Figure S1).

To assess the reproducibility of our gene expression findings, we searched for regulatory patterns that have been previously associated with RPE to neural retina reprogramming. We observed up-regulation of the top five targets previously reported as up-regulated in E4 RPE after 6hPR + FGF2, including *CXCL14, TRIM54, CXCR4, ASL1,* and *FSCN1* (Supplementary Figure S2A) (Tangeman et al., 2021). Similarly, we observed four of the top five down-regulated genes from the same study as also repressed in our dataset, including *ID2, FGFR3, SERPINE3, and PLA2G7*, although we did not observe any change in *CDH5* in response to retinectomy or FGF2 at E4 (Supplementary Figure S2B). Interestingly, all of these targets displayed the expected pattern of regulation in response to retinectomy and FGF2 treatment at E5, pointing toward commonalities in the injury and FGF2 response across the E4 to E5 developmental window. To further understand the observed changes in gene expression, we performed ATAC-seq on nuclei derived from developing E4 and E5 RPE, as well as E4 and E5 RPE 6hPR + FGF2 (Figure 1G). Hierarchical clustering of ATAC signal using all genomic peak regions revealed that samples cluster primarily by developmental or 6hPR + FGF2 conditions, underscoring dynamic chromatin accessibility patterns elicited by retinectomy and FGF2 treatment (Figure 1I). In addition, the generated ATAC-seq data displayed appropriate sequencing quality metrics, accessibility enrichment proximal to TSSs, and high signal-to-noise (Supplementary Figure S3). In summary, our RNA-seq and ATAC-seq datasets capture known features of RPE reprogramming and provide further insight to the underlying chromatin organization.

**FIGURE 2:**
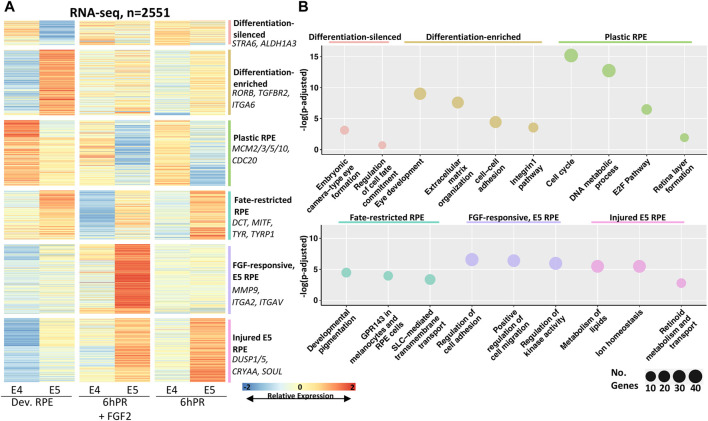
Clustering of differentially expressed genes reveals expression programs associated with RPE commitment and reprogramming. **(A)** Affinity propagation clustering was performed on all genes that are differentially expressed across the E4 to E5 window by the criteria |LFC| ≥ 1 and adj. *p*-value ≤ 0.05 (*n* = 2551), resulting in six clusters. Key genes are summarized on the right of heatmap. The heatmap intensity displays row-normalized gene counts. **(B)** Bubble charts display select gene ontology terms associated with each cluster. Adj. *p*-value is plotted on *y*-axis and the bubble size is proportional to number of terms identified in the analysis.

We next sought to examine the predominant changes in gene expression that define RPE differentiation across the E4 to E5 developmental window and glean insights to the mechanisms by which neural competence is restricted. To this end, we performed pairwise differential gene expression testing of each condition across the E4 to E5 stages (Supplementary File S2) and plotted the relative expression of all differentially expressed genes (DEGs) that exhibit stage-associated regulation at a cut-off of |log fold change| (LFC) ≥ 1 and an adj. *p*-value ≤ 0.05 (Figure 2A). Using affinity propagation clustering, we clustered 2551 total DEGs into six clusters of expression across the tested conditions (Figure 2A, Supplementary Figure S2C, Supplementary File S3). The resolved clusters exhibit expression patterns that point toward potential functions in determining RPE plasticity, so we employed pathway enrichment analysis to determine the associated biological significance (Figure 2B). Two clusters were preferentially depleted or enriched in the developing E5 RPE, which were termed differentiation-silenced or differentiation-enriched, respectively. Pathways associated with differentiation-silenced genes included embryonic eye formation and regulation of cell fate commitment. In contrast, differentiation-enriched genes were associated with eye development and extracellular matrix (ECM) function, including the integrins and cell-cell adhesion, implicating ECM changes as a key feature of RPE differentiation. Two clusters were observed to be preferentially enriched in either E4 or E5 RPE regardless of treatment, which were termed as “plastic RPE” and “fate-restricted RPE” clusters, respectively. Pathways observed in the plastic E4 RPE were dominated by terms suggesting increased proliferation, including cell cycle, DNA metabolic process, and the E2F pathway. Conversely, pathways associated with fate restricted RPE suggested that E5 RPE undergoes significant increases in gene expression related to functional characteristics, including pigment synthesis, melanosome function, and genes encoding solute carrier (SLC) proteins. Finally, the last two clusters were enriched preferentially in the E5 RPE in either injured (6hPR) or FGF2-treated conditions, suggesting that E5 RPE could adopt novel treatment responses to retinectomy and FGF2. Injury-dependent pathways in the E5 RPE included the regulation of cell adhesion and migration, as well as regulation of kinase activity, which could reflect genes with the potential to influence intracellular signal transduction (Supplementary File S3). In contrast, genes associated with FGF2 treatment in E5 RPE pertained largely to lipid metabolism, ion homeostasis, and retinoid metabolism. Collectively, the identified gene clusters point toward significant increases in RPE maturation at E5. These identified gene programs reflect mature RPE function and behavior, including ECM alterations, the dampening of proliferation, increased pigment production, and retinoid metabolism.

### Retinectomy and FGF2 can Activate Initial Signatures of RPE Reprogramming Past the Window of Lineage Restriction

**FIGURE 3:**
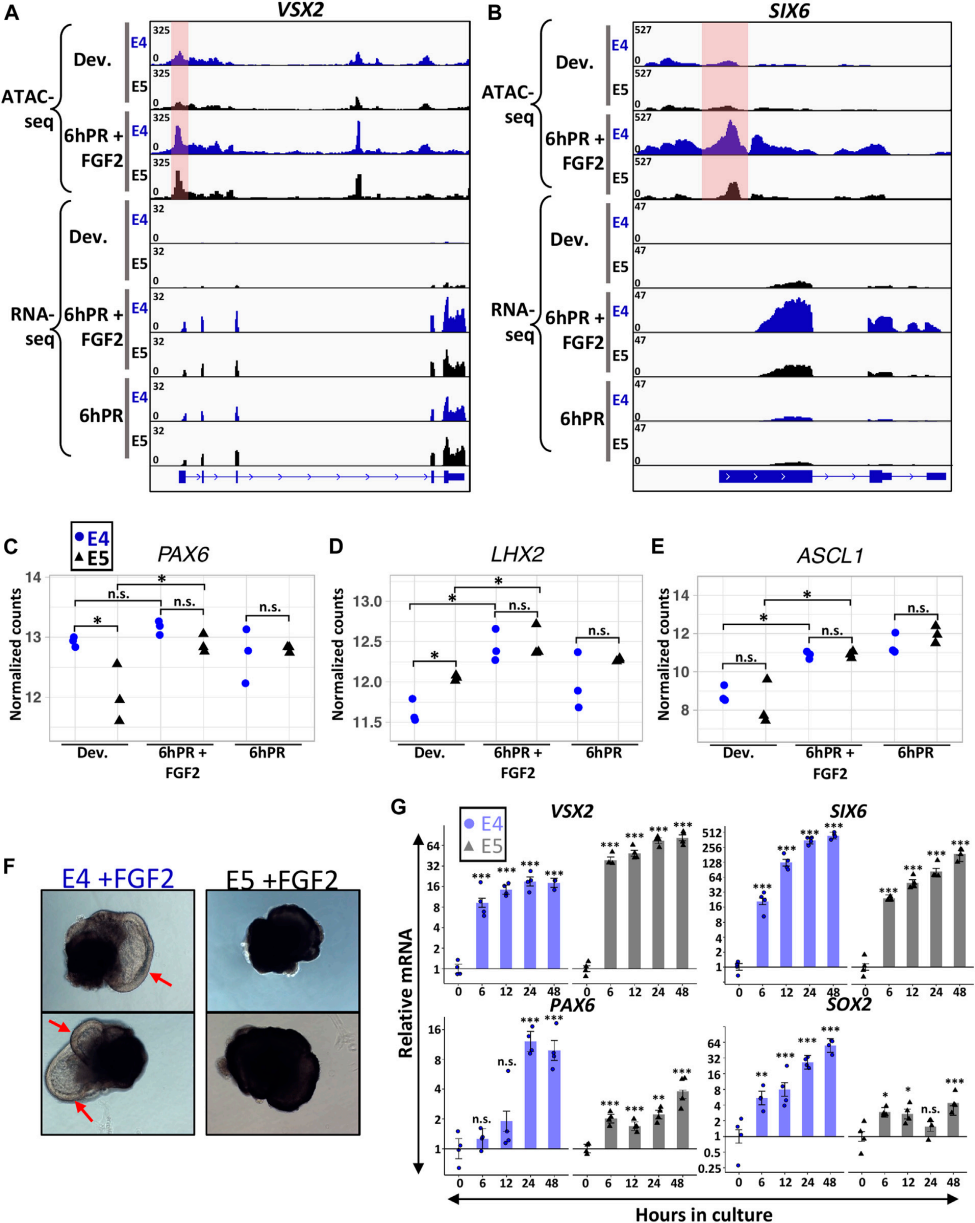
Neural retina identity genes are active 6hPR + FGF2 at E4 and E5. Genome browser views display the *VSX2* **(A)** and *SIX6* **(B)** loci, with the normalized counts for ATAC-seq and RNA-seq assays summarized in the coverage tracks above. The highlighted pink area represents changes in promoter-region accessibility. Normalized gene expression counts are displayed for neural retina-associated genes *PAX6* **(C)** *LHX2* **(D)** and *ASCL1* **(E)**. The *y*-axes are log-transformed. Asterisk (*) denotes an Adj. *p*-value ≤ 0.05. n.s., not significant. **(F)** Two representative RPE explants are shown after 48 h in culture in the presence of FGF2 at E4 (left) or E5 (right). Red arrows point to RPE-derived neural retina from E4 explants. **(G)** Gene expression was measured *via* RT-qPCR using explants cultured in the presence of FGF2 at E4 or E5 and collected at the specified time points. Bar chart displays the average expression, and the error bars represent standard error of the treatment mean based on ANOVA. Each dataset is normalized to basal levels observed at time 0; *y*-axes are log_2_-transformed. qPCR significance: n.s. denotes not significant, * denotes *p*-value < 0.05, ** denotes *p*-value < 0.01, *** denotes *p*-value < 0.001.

Embryonic RPE reprogramming has been shown to initiate with the activation of neural retina genes *VSX2* and *ASCL1*, as well as eye field transcription factors such as *PAX6*, *LHX2*, and *SIX6* (Luz-Madrigal et al., 2014; Tangeman et al., 2021). As such, we sought to determine if the loss of neural competence from the E5 RPE may reside in a failure to activate genes necessary for neural retina fate. *VSX2* is one of the earliest markers to become activated in neural retina progenitors and delineates them from their RPE counterparts (Liu et al., 1994; Fuhrmann, 2010). As expected, we observed the robust activation of *VSX2* in E4 RPE 6hPR + FGF2, as well as 6hPR in the absence of FGF2 treatment, as retinectomy alone has been shown to be sufficient to activate *VSX2* (Figure 3A) (Luz-Madrigal et al., 2014; Tangeman et al., 2021). Interestingly, *VSX2* was expressed at similar levels in E4 and E5 RPE at 6hPR + FGF2, suggesting that *VSX2* activation is not restricted concomitantly with RPE fate restriction. *VSX2* accessibility paralleled our gene expression observations, with dramatically increased promoter accessibility observed after 6hPR + FGF2 in both the E4 and E5 RPE (Figure 3A, shaded pink). In addition, the eye field transcription factor *SIX6* was up regulated in E4 RPE after 6hPR + FGF2, consistent with our previous report (Figure 3B) (Luz-Madrigal et al., 2014). *SIX6* expression in the 6hPR + FGF2 samples was higher at E4 than at E5 (Figure 3B), although the statistical evidence for the increase in mean expression was modest (LFC = −1.44, adj. *p* = 0.1). Similar to *VSX2*, *SIX6* promoter region accessibility tracked closely with our observed changes in expression, with increased accessibility in the E4 RPE after 6hPR + FGF2, and slightly increased accessibility in the E5 RPE after 6hPR + FGF2 (Figure 3B, shaded pink). *PAX6*, which is expressed highly in the neural retina but also is expressed at lower levels in the RPE (Bharti et al., 2012), did not increase in expression in E4 RPE 6hPR + FGF2 when compared to the developing E4 RPE (Figure 3C). In contrast, *PAX6* was expressed lower in the developing E5 RPE relative to E4 levels but increased in the E5 RPE after 6hPR + FGF2 to similar levels as observed at E4 (Figure 3C). Additionally, *LHX2* and *ASCL1* were activated to similar levels in both the E4 and E5 RPE after 6hPR + FGF2, and also showed increases in response to 6hPR alone at both stages (Figures 3D,E). Altogether, our observed gene expression changes suggest that genes encoding neurogenic transcription factors exhibit similar regulatory patterns in response to retinectomy and FGF2 treatment in both the E4 and E5 RPE.

In order to explore stage-specific regulation of neural retina identity factors past 6 h, we turned to an RPE explant culture system that mirrors the *in vivo* reprogramming phenotype in an FGF2-dependent manner (Sakami et al., 2008). E4 RPE explants cultured in the presence of FGF2 show clear neural retina domains within 48 h of culture, whereas no neural morphology is observed using E5 RPE explants cultured for 48 h with FGF2 (Figure 3F). In addition, no neural features are observed at either stage when cultured in the absence of FGF2 for 48 h (Supplementary Figure S4A). Using this set-up, we monitored the expression of neural retina factors *VSX2*, *SIX6,* and *PAX6 via* RT-qPCR by collecting either E4 or E5 explants at 6,12, 24, and 48 h in culture (Figure 3G). In agreement with our *in vivo* observations, we observed potent up regulation of *VSX2* in both the E4 and E5 RPE explants as early as 6 h of culture, and *VSX2* levels remained increased at both stages through 48 h. Similarly, we observed up-regulation of *SIX6* after 6 h of culture using E4 RPE explants. However, in contrast to our *in vivo* results, we were able to observe strong evidence for elevated *SIX6* expression using E5 RPE explants, and this high level of expression was maintained through 48 h of culture. Quantification of *PAX6* further validated our RNA-seq results, in which we did not observe evidence of up-regulation after 6 h of culture using E4 RPE explants, but we did observe a slight increase in expression after 6 h using E5 RPE explants. However, the E4 RPE explants underwent a dramatic increase in *PAX6* levels after 24 h of culture, and displayed a regulatory pattern distinctly more robust than observed in E5 RPE. Notably, our qPCR results for *PAX6* in the E5 explants displayed heteroscedasticity (Levene’s test α = 0.01), although the nonparametric Dunn’s test still indicated evidence of difference from control (*p* < 0.05) at 24 and 48 h in culture (Supplementary File S1). As expected, the neural progenitor marker *SOX2* was robustly elevated in the E4 RPE explants early during culture, and although we observed small increases in *SOX2* expression at several time points using E5 RPE explants, they differed from our observations at E4 by several orders of magnitude. Collectively, these observations reinforce the concept that the neural retina factors *VSX2* and *SIX6* remain FGF2-inducible past the window of RPE lineage restriction. However, it is possible that *PAX6*, which has context-specific roles that delineate its RPE and neural retina functions (Bharti et al., 2012; Raviv et al., 2014), could be regulated during later stages of RPE reprogramming to influence neural competence.

### RPE Enters Stable Proliferative Quiescence Concomitant With the Loss of Neural Competency

**FIGURE 4:**
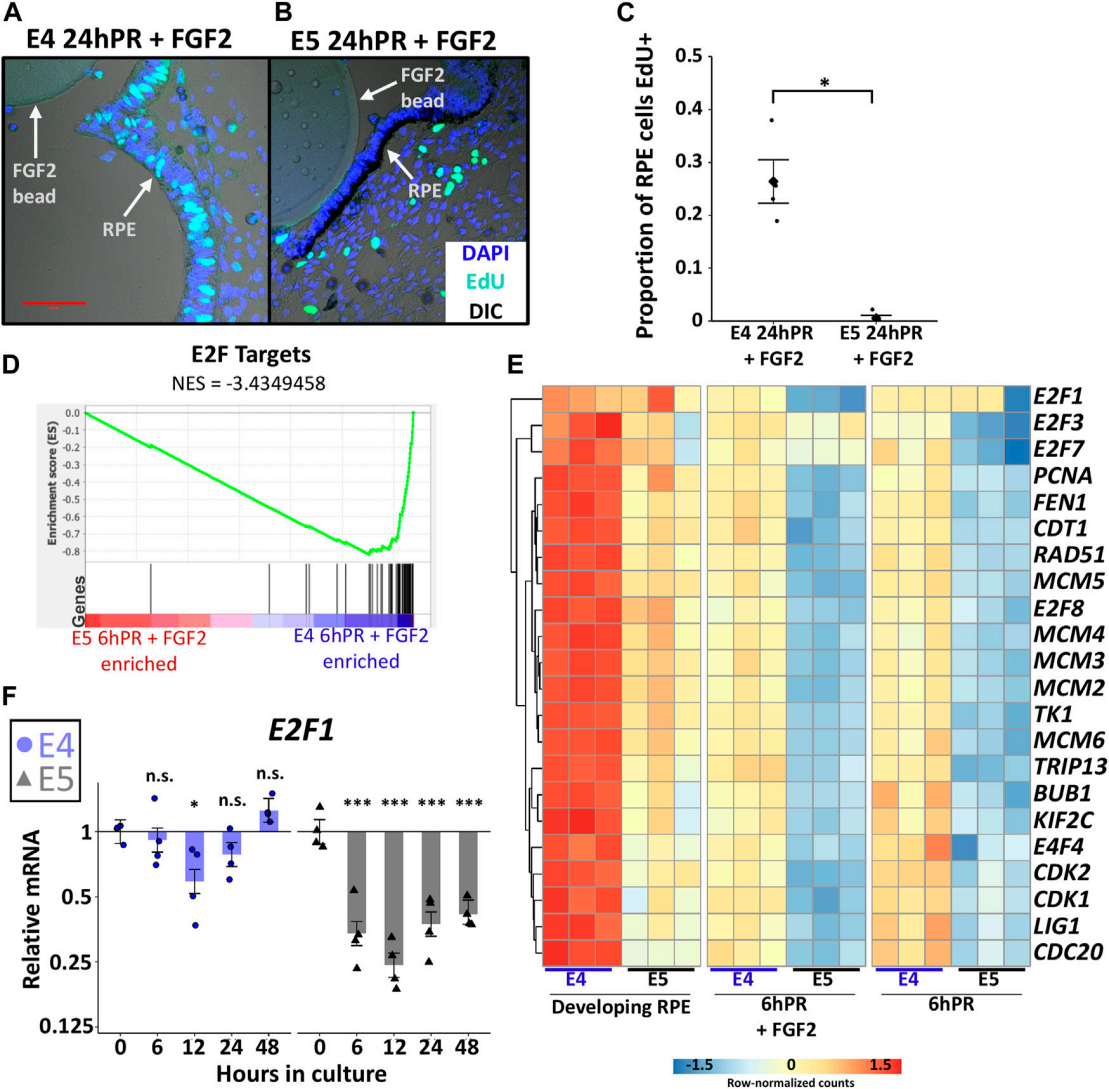
Proliferation is tightly regulated across the window of RPE cell fate restriction. **(A)** EdU and DAPI fluorescence staining of embryo sections collected 24 h PR (24hPR) and FGF2 treatment was performed at either E4 **(A)** or E5 **(B)**. Fluorescence channels are overlayed with differential interference contrast (DIC) image. 50 μm scale bar in **(A)** applies to both panels. Chart displays the proportion of EdU positive RPE cells observed 24hPR + FGF2 at E4 and E5 **(C)**. Diamond represents mean proportion; asterisk (*) denotes Adj. *p* value <0.05. **(D)** Gene set enrichment analysis (GSEA) was performed on normalized RNA-seq expression values from 6hPR + FGF2 E4 and E5 samples. A low normalized enrichment score (NES = −3.4349458) for E2F targets indicates high enrichment in the E4 6hPR + FGF2 RPE and depletion from the E5 6hPR + FGF2 RPE. Each black vertical bar represents a gene in the pathway. **(E)** The row-normalized heatmap displays RNA-seq expression values for E2F targets genes and proliferation-associated factors. **(F)** RT-qPCR was used to measure *E2F1* expression in E4 and E5 explants cultured in the presence of FGF2 and collected at the specified time points. Bar chart displays the average expression value, and the error bars represent standard error of the treatment mean based on ANOVA. Each dataset is normalized to basal levels observed at time 0; *y*-axes are log_2_-transformed. qPCR significance: n.s. denotes not significant, * denotes *p*-value < 0.05, ** denotes *p*-value < 0.01, *** denotes *p*-value < 0.001.

Mature RPE remains mitotically quiescent throughout the entire lifetime of an individual, but RPE cells can be coaxed to divide *in vitro* under the direction of growth factors (Stern and Temple, 2015). It is not known whether proliferation is required for RPE to neural retina reprogramming, although cell type specification during neural retina development is highly coordinated with cell cycle timing (Dyer and Cepko, 2001). A study of the initial non-proliferative stage of embryonic RPE reprogramming (6hPR) uncovered that RPE undergoes a transient repression of cell cycle genes in response to retinectomy and FGF2 treatment (Tangeman et al., 2021). However, by 24 h PR and FGF2 treatment, dedifferentiated RPE cells become BrdU positive and p27kip1 negative, and begin proliferating to form a neuroepithelium (Luz-Madrigal et al., 2014). Given our observations, we sought to determine if proliferative status could be associated with the loss of neural competency from E5 RPE. Using EdU incorporation to measure S-phase entry, we observed that E5 RPE 24 h PR and with FGF2 treatment resulted in almost complete absence of EdU+ RPE cells compared to the equivalent E4 RPE (Figures 4A–C). EdU staining performed using the developing (untreated) posterior RPE revealed that S-phase entry was progressively reduced from E3 to E5 of development, coinciding with the loss of neural competence (Supplementary Figure S4B,C). Thus, the basal proliferative status of the developing RPE strictly correlates with the observed ability for RPE to undergo FGF2-induced proliferation and reprogramming.

We next sought to characterize the observed proliferation patterns in the context of gene expression changes. Gene set enrichment analysis (GSEA) of our samples revealed a consistent enrichment of the term “E2F target genes” in E4 RPE relative to E5 RPE using both developing and 6hPR + FGF2 conditions (Figure 4D and Supplementary Figure S4D). The E2F family of transcription factors are master regulators of cell cycle progression, and the activity of E2F can be assayed *via* the expression of E2F target genes (Kent and Leone, 2019). To further break down the transcriptional control of cell cycle regulators during early RPE reprogramming, we plotted the expression of known E2F targets and related cell cycle mediators (Figure 4E). Largely, the observed trends echoed previous findings that cell cycle related gene expression is repressed in E4 RPE after 6hPR + FGF2, during the initial non-proliferative phase (Tangeman et al., 2021). However, our novel findings reveal that this trend is exacerbated in E5 RPE, as the E5 RPE exhibits lower basal levels of cell cycle transcripts that are further repressed following retinectomy or FGF2 treatment. Interestingly, the E2F factors *E2F1*, *E2F2*, and *E2F7* each contributed to this trend with slightly different expression dynamics (Figure 4E). To further observe E2F dynamics past 6 h, we performed RT-qPCR to measure E2F dynamics through 48 h using our E4 and E5 RPE explant system. Similarly, we observed an initial slight repression of *E2F1* expression within 12 h of E4 explant culture in the presence of FGF2, but expression was recovered by 48 h (Figure 4F). In contrast, *E2F1* expression was rapidly repressed following the culture of E5 explants with FGF2, and *E2F1* repression was maintained through 48 h of culture (Figure 4F). Together, our results point toward a model in which RPE cells stably exit the cell cycle as development progresses, between E4 and E5. Following this restriction point, RPE cells cannot be induced to proliferate by retinectomy and FGF2 treatment, and the E2F family of cell cycle regulators are likely to play an instrumental role in the loss of proliferative capacity. Thus, it is possible that proliferation is a necessary requisite for RPE to neural retina reprogramming, although it should be noted that in some contexts E2F factors can induce RPE proliferation in the absence of a change in cell identity (Kampik et al., 2017).

### Broad Attributes of RPE Maturation Are Enhanced in the E5 RPE Independently of Retinectomy and FGF2 Treatment

**FIGURE 5:**
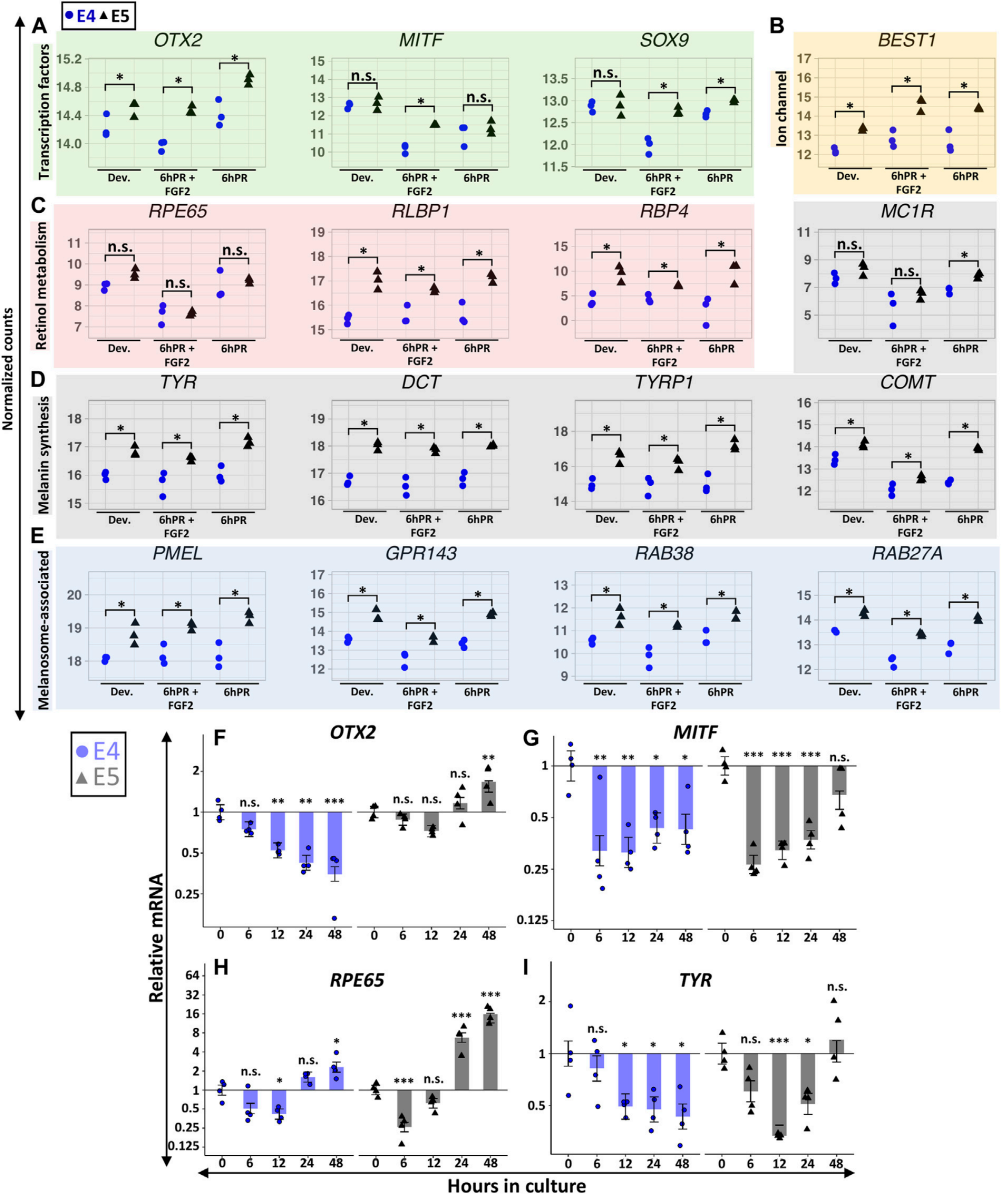
Gene signatures associated with RPE maturation are broadly elevated in the E5 RPE. Normalized RNA-seq expression values are displayed for genes encoding RPE-associated transcription factors **(A)**, the ion channel *BEST1* **(B)**, retinol metabolism and transport factors **(C)**, melanin synthesis machinery **(D)**, and melanosome-associated proteins **(E)**. For **(A**–**E)**, asterisk (*) denotes an Adj. *p*-value ≤ 0.05 and n.s., not significant. Relative gene expression was measured in RPE explants using RT-qPCR, and is displayed for *OTX2* **(F)**, *MITF* **(G)**, *RPE65* **(H)**, and *TYR* **(I)**. Bar chart displays the average expression, and the error bars represent standard error of the treatment mean based on ANOVA. Each dataset is normalized to basal levels observed at time 0; *y*-axes are log_2_-transformed. qPCR significance: n.s. denotes not significant, * denotes *p*-value < 0.05, ** denotes *p*-value < 0.01, *** denotes *p*-value < 0.001.

Embryonic RPE reprogramming initiates with the simultaneous activation of neural retina gene programs and the repression of RPE identity factors, and these changes are transcriptionally detectable within the first 6 h following retinectomy and FGF2 treatment. Our initial results indicated that the E5 RPE can be induced to express key genes associated with neural retina fate (Figure 3), so we next asked if RPE dedifferentiation may be hindered by a failure to efficiently repress RPE identity factors. Indeed, our RNA-seq observations suggested that E5 RPE was consistently enriched for numerous factors associated with functional characteristics of mature RPE, in many cases independently of retinectomy or FGF2 treatment. The RPE determining transcription factor *OTX2* was enriched in E5 RPE relative to E4 RPE across all tested conditions (Figure 5A). Similarly, the pigmentation regulator *MITF* was repressed by retinectomy and FGF2 treatment at both stages but remained elevated at E5 relative to E4 levels. A similar pattern was observed for *SOX9*, which regulates visual cycle gene expression (Masuda et al., 2014), as we detected elevated *SOX9* levels in E5 RPE in both the 6hPR and 6hPR + FGF2 conditions. This pattern extended to other broad factors that are directly involved with RPE function, including the ion channel *BEST1* (Figure 5B) and retinol metabolism genes *RLBP1* and *RBP4* (Figure 5C). Interestingly, we did not detect an evident difference in *RPE65* expression (Figure 5C), although *RPE65* may be associated with RPE function at later stages of maturation (Petrus-Reurer et al., 2021). In addition, genes associated with pigment synthesis, including the paralogous eumelanin synthesis genes *TYR*, *DCT*, and *TYRP1*, as well as the catecholamine methyltransferase *COMT*, were consistently enriched in E5 RPE across the observed conditions. In contrast, the melanocortin receptor *MC1R* was enriched in E5 RPE, but only in the 6hPR condition (Figure 5D). Genes encoding the melanosome-associated proteins *PMEL*, *GPR143*, *RAB38*, and *RAB27A* were all consistently up-regulated in E5 RPE across all tested conditions (Figure 5E). More broadly, this pattern could be observed in our GSEA results, as genes associated with fetal RPE development were among the top enriched gene sets in the E5 RPE in both the developing and 6hPR + FGF2 conditions (Supplementary Figures S5A,B). Of note, we also observed extensive enrichment of integrins and related ECM components in the E5 RPE (Supplementary Figures S5C,D). The ECM composition of the RPE basal membrane, Bruch’s membrane, is directly related to RPE maturation and function, and RPE reprogramming is mediated by ECM interactions in some contexts (Reh et al., 1987; Benedicto et al., 2017).

In contrast to the dynamic regulation of promoter accessibility observed for neural retina associated genes (Figures 3A,B), the promoter accessibility of RPE maturation genes generally underwent comparatively subtle changes (Supplementary Figure S5E). Promoter accessibility of *OTX2* showed modest enrichment in E5 samples relative to E4 samples, and similarly, we observed reduced basal levels of *MITF* promoter accessibility in the 6hPR + FGF2 samples at both E4 and E5. The *SOX9* promoter demonstrated marginally reduced accessibility specific to the E4 6hPR + FGF2 samples, corresponding to the observed changes in gene expression. Interestingly, while we did not observe basal differences in *MC1R* gene expression between the developing E4 and E5 RPE, we did find evidence that *MC1R* promoter accessibility was significantly higher in the E5 RPE. Moreover, *MC1R* underwent a pronounced loss of accessibility at both stages following 6hPR + FGF2 treatment, although any significance of the discrepancy between *MC1R* promoter accessibility and expression in the context of RPE neural competency is still unclear. Both the *TYRP1* and *GPR143* promoters paralleled gene expression changes, demonstrating increased accessibility in E5 relative to E4 conditions. Thus, the promoter accessibility of RPE maturation factors appears to be highly correlated with transcriptional output and may serve as a basis for understanding the stage-specific gene expression observed in RPE cells. These relatively subtle patterns could be explained by a tendency for the accessibility of regulatory elements to precede expression changes, as accessibility changes in the developing E4 RPE could foreshadow expression dynamics observed at E5.

To contextualize these stage-specific regulatory patterns past the first 6 h of reprogramming, we again turned to our *ex vivo* explant culture system with FGF2. *OTX2* expression at E4 was similar to the initial regulatory pattern observed *in vivo*, with strong evidence for a decrease in expression observable within 12 h, and which continued through 48 h (Figure 5F). In E5 RPE explants, *OTX2* expression increased from basal levels by 48 h. Thus, our *ex vivo* observations reinforce and extend our *in vivo* findings to suggest that *OTX2* expression is not efficiently silenced by FGF2 by E5, whereas *OTX2* is readily repressed at E4. Interestingly, *MITF* expression rapidly decreased in both E4 and E5 explants, similar to the repression pattern observed *in vivo* (Figure 5G). However, *MITF* expression remained repressed through 48 h in the E4 explants, whereas *MITF* expression in E5 explants returned to levels similar to the initial basal expression by 48 h (*p* = 0.12). We observed potential heteroscedasticity present across groups for *MITF* expression at E5 (Levene’s test α = 0.04), although Dunn’s nonparametric test still provided evidence for a transitory repression of *MITF* at 6 and 12 h in culture (Supplementary File S1). *RPE65* was also transiently repressed in both the E4 and E5 explants, but expression recovered to basal levels by 24 h and continued to increase (Figure 5H). Similar to *OTX2* and *MITF*, *TYR* underwent stage-specific regulation, and after undergoing transient repression in both E4 and E5 explants, *TYR* levels returned to basal levels by 48 h in E5 explants only (Figure 5I). Our observations for *TYR* in E5 explants displayed evidence of heteroscedasticity as well (Levene’s test α = 0.02), and subsequent analysis with Dunn’s test produced evidence for repression of TYR at 12 h in culture (adj. *p* = 0.013; Supplementary File S1), but not at the other observed time points.

### Chromatin Accessibility Is Globally Remodeled During RPE Lineage Commitment and Perturbs Underlying Transcription Factor Regulatory Networks

**FIGURE 6:**
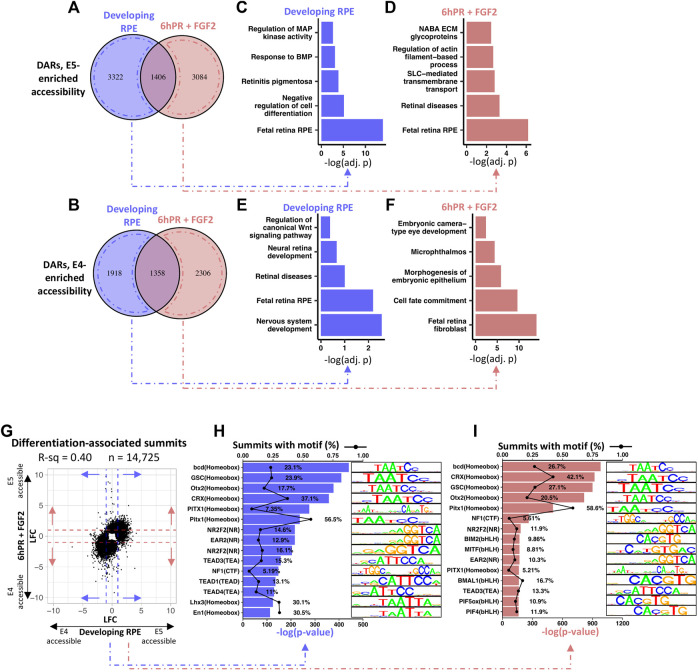
RPE maturation is accompanied by differential accessibility at retinal development genes and homeobox transcription factor binding sites. **(A)** Venn diagram summarizes the overlap of up-regulated DARs (DARs with increased E5 accessibility) between the developing and 6hPR + FGF2 conditions, defined by a cut-off of Adj. *p*-value ≤ 0.05. **(B)** Similarly, Venn diagram displays down-regulated DARs (E4-enriched DARs) for the two conditions. DARs unique to either condition were annotated to the nearest gene TSS within 10 kilobases, and pathway enrichment analysis was performed for DARs enriched in the developing E5 RPE **(C)**, the E5 6hPR + FGF2 RPE **(D)**, the developing E4 RPE **(E)**, or the E4 6hPR + FGF2 RPE **(F)**. **(G)** The log fold change (LFC) of differentially accessible peak summits between the E4 and E5 conditions are plotted. Dashed lines represent a LFC cut-off of 1 that was used to select summits for motif analysis. HOMER motif detection software was used to identify transcription factor DNA motifs overrepresented in the summits of peaks differentially accessible across the developing **(H)** or 6hPR + FGF2 **(I)** RPE, and the top 15 enriched motifs are shown.

We next set to define the regulatory landscape of developing RPE from the perspective of stage-specific changes in accessibility. Analysis of differentially accessible regions (DARs) in the E4 RPE compared to the E5 RPE revealed a total of 8,004 and 8,154 DARs in the developing and 6hPR + FGF2 conditions, respectively (Supplementary File S4). Breakdown of these regions by their regulatory pattern revealed 4,728 DARs and 4,490 DARs with elevated accessibility at E5 in the developing and 6hPR + FGF2 conditions, respectively (Figure 6A). Similarly, we observed a respective 3,276 and 3,664 DARs with elevated accessibility at E4 in the developing and 6hPR + FGF2 conditions (Figure 6B). To assess whether DARs can be associated with biological function, we grouped DARs by their observed regulatory pattern and performed pathway enrichment analysis using the nearest genes within 10 kilobases. Interestingly, the DARs with enhanced enrichment in the developing E5 RPE were enriched proximal to genes associated with MAPK and BMP signaling, retinitis pigmentosa, cell differentiation, and fetal RPE (Figure 6C). Moreover, analysis of the DARs with enhanced E5 accessibility in the 6hPR + FGF2 RPE revealed genes associated with ECM glycoproteins, actin-based processes, SLC transporters, retinal diseases, and also fetal retina RPE (Figure 6D). In contrast, analysis of the genes associated with DARs that are more accessible in the developing E4 RPE returned pathways including WNT signaling, neural retina development, retinal disease, fetal retina RPE, and nervous system development (Figure 6E). Finally, DARs with accessibility uniquely enriched in the reprogramming (E4 6hPR + FGF2) RPE were associated with embryonic camera-type eye development, microphthalmos, epithelium morphogenesis, cell fate commitment, and fetal retina fibroblasts (Figure 6F). Next, to determine if the observed accessibility patterns broadly relate to gene expression, we binned all transcripts into quintiles by basal expression levels and plotted the measured accessibility within 2 kilobases of each TSS (Supplementary Figure S6A). We observed a positive correlation between basal gene expression and promoter region accessibility, underscoring the agreement between our RNA-seq and ATAC-seq values and adhering to published observations that promoter region accessibility has a positive correlation with transcriptional output (Starks et al., 2019). To further determine the genomic distribution of these DARs, we categorized the observed accessibility patterns by their overlapping genomic features (Supplementary Figure S6B). Interestingly, a large proportion of the accessibility changes were found in intronic and distal intergenic regions, suggesting the potential for the DARs to coincide with distal regulatory elements, such as transcription factor-bound cis-regulatory elements.

To determine the potential for accessibility changes to modify transcription factor binding sites, we plotted the relative accessibility of the peak region of all significantly altered DARs across the developing and 6hPR + FGF2 conditions (Figure 6G). Peak summits with an accessibility |LFC| ≥ 1 across either the developing or 6hPR + FGF2 conditions were scanned for overrepresentation of transcription factor motifs using the HOMER motif discovery software. Differentially accessible peak summits in the developing RPE were found to be highly enriched for the TAATCC homeobox motif, which is recognized by the DNA-binding homeodomain of a number of homeobox factors that regulate eye development, including OTX2, CRX, and PITX1 (Figure 6H). Moreover, we observed a high overrepresentation of motifs resembling the TEA motif recognized by TEAD1, TEAD3, and TEAD4, which is notable given the known roles for TEAD transcription factors in governing RPE specification and function (Miesfeld et al., 2015; Buono et al., 2021). Moreover, the NR motifs recognized by NR2F1 and NR2F2 were highly overrepresented in the observed DARs, which are known to regulate RPE vs neural retina identity throughout the differentiating optic vesicle (Tang et al., 2010). Similarly, the accessibility changes observed in the RPE samples 6hPR + FGF2 were also highly enriched for the homeobox binding motif (Figure 6I). However, amongst the 6hPR + FGF2 responsive peaks, we additionally observed high enrichment of the bHLH motif, which includes the MITF binding motif. This observation in accessibility changes can be reconciled with our *in vivo* observations of *MITF* expression, as we only observed stage-specific differences in *MITF* following 6hPR + FGF2, but not the developing (untreated) RPE (Figure 5A). Finally, the stage-specific accessibility changes in RPE 6hPR + FGF2 are also highly associated with TEA and NR binding motifs. Together, these observations suggest that RPE lineage commitment occurs concurrent to significant regulatory changes at key transcription factor binding sites that are known to regulate RPE identity and function.

In addition to stage-specific accessibility patterns, we also observed a large number of DARs that underwent accessibility changes in response to retinectomy and FGF2 treatment. A total of 61,427 peak summits exhibited differential accessibility when comparing developing RPE to 6hPR + FGF2 RPE at either E4 or E5 (Supplementary Figure S6C). Interestingly, a high agreement between accessibility changes were observed when comparing the treatment response observed in the E4 and E5 RPE (R-sq = 0.76), suggesting that peaks responsive to retinectomy and FGF2 treatment largely behave concordantly regardless of developmental stage. We then searched the responsive peak summits for the overrepresentation of motifs with the HOMER software. Amongst the top identified motifs in both the E4 and E5 RPE was the motif recognized by CTCF (Supplementary Figures S6D,E). Interestingly, amongst the top overrepresented motifs were also numerous factors containing the bZIP domain, such as AP-1 family members. A number of AP-1 factors have been previously shown to be transcriptionally regulated during the early stages of E4 RPE reprogramming in either retinectomy- or FGF2-dependent manners (Tangeman et al., 2021). Similarly, we observed elevated expression of both *JUN* and *FOS* after 6hPR + FGF2 in both the E4 and E5 RPE (Supplementary Figure S6F). Together, these observations suggest the AP-1 transcription factors are regulated at the transcriptional level in response to retinectomy and FGF2 treatment, and their accessibility footprint can be observed in both plastic E4 and restricted E5 RPE. Future work may determine if downstream functions of AP-1 factors are altered across this developmental window to influence RPE neural competence.

Finally, intrigued by our observations suggesting that specific transcription factors are regulating RPE neural competence (Figures 6H,I), we sought to determine if we could further parse our findings to glean insights into the regulatory logic of the participating transcription factors. To accomplish this, we assigned each of the differentially accessible peak summits to the nearest gene TSS and retained only regions that are associated with a significant change in gene expression from our RNA-seq observations (Supplementary Figure S7). Next, we categorized each of the observations based on the directionality of the accessibility change and the gene expression change, and separately performed HOMER motif discovery analysis on each cohort of loci. Within our developing RPE samples, we observed an overrepresentation of PITX1:Ebox and NR2F2 motifs in regions with higher E4 accessibility than E5 accessibility, and these regions were associated with both increases and decreases in gene expression (Supplementary Figure S7A). In contrast, the regions with higher accessibility at E5 were dominated by TEAD motifs, which were both positively and negatively associated with expression changes. We also observed overrepresentation of the similar BCD, GSC, and OTX2 motifs in both E4- and E5-accessible regions, although these regions were clearly biased toward E5 accessible regions that are associated with increases in gene expression. Importantly, we further observed evidence for overrepresentation of the NF1 motif in E5-accessible regions that are associated with a decrease in expression, suggesting that this factor may play a role in gene silencing in the E5 RPE. Combined analysis of gene expression and accessibility in the RPE following 6hPR + FGF2 treatment also identified NR2F2 and PITX1:Ebox motifs associated with E4-accessible regions, which were again associated with both increased and decreased gene expression (Supplementary Figure S7B). However, in the E4-accessible regions in 6hPR + FGF2 RPE, we further observed overrepresentation of the RARa and ESRRB motifs, which were associated with decreased expression, and the LHX1/3 motifs, which were associated with increased expression. On the other hand, E5 accessible regions in the 6hPR + FGF2 treated RPE were largely overrepresented for homeobox motifs, including OTX2. Of these regions, OTX2 was strongly overrepresented in the regions associated with increased gene expression, although notably did also show modest enrichment amongst regions associated with gene silencing. Finally, amongst the E5 accessible regions in the 6hPR + FGF2 RPE, we further observed enrichment of the MITF motif amongst regions associated with increased expression, and overrepresentation of the NFY motif in regions associated with decreased expression. It should be noted that the association between differential accessibility and changes in expression of proximal genes are not always directly linked, but this analysis may serve as a starting point for future experimentation aimed at resolving the gene regulatory networks that determine RPE neural competence.

### Modification of OTX2 and Broader Homeobox Transcription Factor Binding Sites Are Defining Features of RPE Lineage Commitment

**FIGURE 7:**
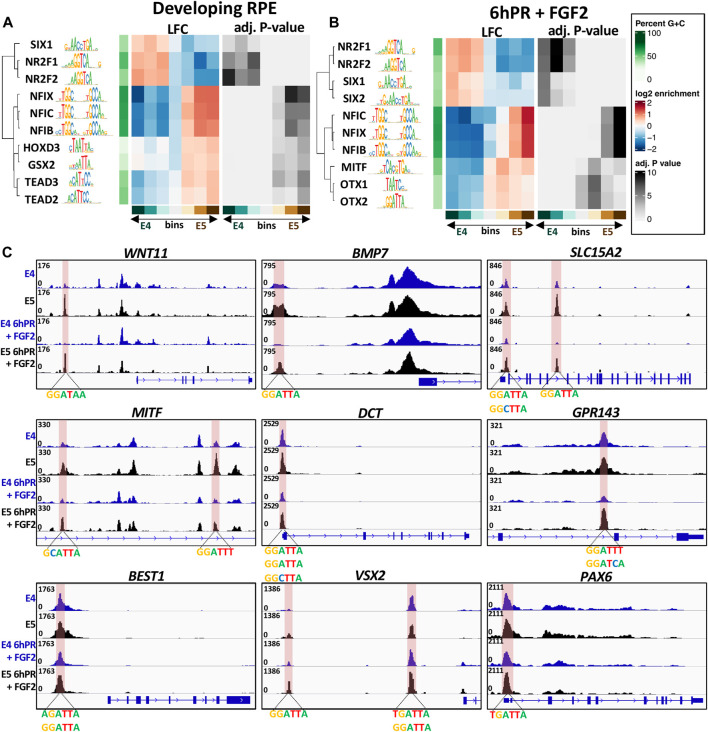
Overrepresented motifs in open regions of the E4/E5 RPE. The monaLisa motif analysis software was used to bin differentially accessible summits by accessibility across the E4 and E5 conditions. Each bin was analyzed for the overrepresentation of transcription factor motifs found in the JASPAR 2022 database. Enriched motifs identified at −log(p-Adj.) >3 in any bin were recorded and select factors associated with the developing RPE **(A)** or 6hPR + FGF2 **(B)** samples were plotted. **(C)** Genome browser displays ATAC-signal proximal to DARs containing the OTX2 binding motif. The region of interest is highlighted in pink and associated motifs are summarized below the track.

Given the observed motif enrichment patterns, we next asked if any of these motifs were preferentially accessible in E4 or E5 RPE. To answer this question, we first categorized differentially accessible peak summits into seven bins by LFC across the E4 to E5 window. Using the monaLisa motif detection tool, we analyzed each bin for the overrepresentation of transcription factor binding motifs obtained from the JASPAR 2022 vertebrate motif database. First focusing on the developing (untreated) RPE, we observed groups of related transcription factor motifs which were preferentially accessible in either the E4 or E5 RPE (Figure 7A). Interestingly, we observed that NR2F1 and NR2F2 motifs were highly enriched amongst the peaks accessible in E4 RPE and depleted from peaks that are accessible in E5 RPE, suggesting that in general NR2F1/2 binding motifs become less accessible during RPE lineage commitment. Moreover, we observed the opposite trend for motifs for the NFI and TEAD family transcription factors, suggesting that NFI and TEAD binding sites become, in general, more accessible in the E5 RPE. Although we did not find any evidence for overrepresentation of the OTX2 motif in any accessibility bin for the developing RPE, we did observe enrichment of homeobox factors such as HOXD3 and GSX2, which share similar binding motifs to OTX2 (Figure 7A). In general, the observed homeobox factors tended to be enriched in bins with increased accessibility in the E5 RPE.

Shifting focus to the RPE 6hPR + FGF2, we also observed a strong E4 accessibility preference for the NR2F1/2 motifs, and an E5 accessibility preference for NFI motifs (Figure 7B). Similar to previous results, we were able to detect enrichment of the MITF motif only in the ATAC signature of our 6hPR + FGF2, which was revealed to have a strong overrepresentation in E5-accessible regions. Additionally, we observed a preferential accessibility of SIX1 and SIX2 in the E4 RPE relative to the E5 RPE after 6hPR + FGF2. We observed strong evidence for enrichment of the OTX1 and OTX2 binding motif in RPE 6hPR + FGF2, which showed a clear overrepresentation in regions of heightened accessibility in the E5 RPE. The full set of significant transcription factors identified in the developing and 6hPR + FGF2 RPE are provided (Supplementary Figure S8). To further validate these findings, we applied an alternate approach using the monaLisa analysis software in which the accessible regions of interest were scanned for overrepresentation of 6-mer nucleotide sequences, and then the overrepresented nucleotide strings are matched to known transcription factor motifs. Using this method, we were able to identify enrichment of 6-mers that resemble the NR2F1/2, TEAD family, and homeobox family factors in both the intact and 6hPR + FGF2 RPE samples (Supplementary Figure S9).

Given the increased expression of *OTX2* in the E5 RPE (Figures 5A,F), as well as the overall increased accessibility of OTX2 and homeobox motifs in the E5 RPE (Figures 7A,B), we next asked if specific changes in OTX2 motif accessibility could influence RPE neural competence. To address this question, we created a library of all ATAC peak summits that contain the OTX2 binding motif and that undergo a significant change in accessibility (Supplementary File S5). Interestingly, we observed numerous such peaks located proximal to genes known to regulate RPE fate (Figure 7C and Supplementary File S5). One of the identified OTX2 motifs is located directly upstream of *WNT11*, which is a gene known to contribute to early eye morphogenesis (Fuhrmann, 2008). A similar regulatory region was located upstream of *BMP7*, which is highly expressed in the developing RPE (Steinfeld et al., 2017), as well as two additional regulatory regions found within *SLC15A2* introns. We also identified differentially accessible OTX2 motifs near numerous modulators of RPE identity, including two such sites within a *MITF* intron, one site within the *DCT* promoter, one site within an intron of GPR143, and one site directly upstream of *BEST1*. Each of these sites displayed a robust increase in accessibility in the E5 RPE relative to E4 levels in the developing or 6hPR + FGF2 conditions. Finally, we observed two additional OTX2 motifs upstream of the *VSX2* TSS, as well as one within the *PAX6* promoter region, which could point toward a *VSX2* or *PAX6* regulatory element that could be modified to influence regenerative outcome. Collectively, these observations encompass regulatory regions of interest that exhibit increased accessibility in E5 RPE and have the potential to act as binding sites for OTX2 or related homeobox transcription factors. The observed accessibility patterns of these loci, combined with their proximity to known regulators of RPE identity, suggest that these regulatory elements could function as primary barriers to RPE neural competence.

## Discussion

In the present study, we demonstrate that the loss of RPE neural competence coincides with the progressive activation of functional gene sets pertaining to pigment synthesis machinery, retinal metabolism, ion transport, and ECM deposition. Along with these functional attributes, the committed RPE cells exhibit altered regulatory behavior associated with key transcription factors, including *OTX2* expression and OTX2 motif accessibility, when compared to more plastic RPE. Despite these observations, E5 RPE can be induced to express many reprogramming factors at comparable levels to the ones observed in the E4 RPE, including the expression of *VSX2, ASCL1, PAX6* and *LHX2*. These observations suggest that certain features of the neurogenic response elicited by FGF2 remain intact for some time after neural competence is lost, although further studies are needed to determine if these regulatory patterns will be relevant to RPE at later stages of maturity. In this regard, it has been demonstrated using aged human donors that a subpopulation of RPE cells (termed RPE stem cells) retain a capacity for self-renewal *in vitro* (Salero et al., 2012). It was further demonstrated that culture of RPE stem cells in a neuronal differentiation media containing FGF2 was sufficient to activate *LHX2* and *RAX*, although notably the authors also reported up-regulation of *OTX2* and down-regulation of *SIX3* and *PAX6*. In contrast, *in vivo*, mammalian RPE is thought to remain largely non-proliferative, and our results suggest that the onset of proliferative quiescence could be directly tied to terminal differentiation of RPE. A recent study demonstrated that murine RPE can be induced to proliferate and undergo limited self-renewal *in vivo* following the targeted over expression of *E2F2* (Kampik et al., 2017). Taken in light of our findings, E2F factors may serve as a necessary requisite for the induction of neural regeneration from RPE cells. Collectively, it is possible that mature RPE cells may retain some latent features from the embryonic state, but more studies are needed to understand how the associated factors act differently within the embryonic and mature RPE contexts.

Our observations demonstrate that the expression of neural factors such as *VSX2, ASCL1, PAX6* and *LHX2* is insufficient to drive neural progenitor formation from the E5 RPE. Taken in the context of our other observations, it is likely that one or more RPE determining factors act as a counterbalance to these factors in order for the RPE phenotype to be maintained, preventing dedifferentiation. We identify a number of functional pathways that could potentially act to solidify the RPE fate, and we present evidence that OTX2 has the potential to act as a primary determinant in restricting RPE neural competence. OTX2 is an early determinant of the RPE cell fate (Martinez-Morales et al., 2001; Martínez-Morales et al., 2003). Interestingly, OTX2 is also repressed in RPE cells of the newt *Cynops pyrrhogaster* during the process of neural retina regeneration *via* RPE reprogramming (Sakami et al., 2005), suggesting that OTX2 activity may be antagonistic to regeneration. Moreover, the conditional knock-out of OTX2 in adult murine RPE leads to progressive degeneration of photoreceptors, disrupted RPE homeostasis, and altered melanogenesis and retinal metabolism gene networks, underscoring OTX2 as a fundamental factor for the maintenance of RPE cell identity (Housset et al., 2013). In our FGF2-treated explants, we observed transient repression of the RPE factors *OTX2*, *MITF*, *RPE65*, and *TYR*, although the expression of each of these genes was recovered or increased in E5 explants by 48 h of culture. In contrast, each of these genes remained repressed in the E4 explants through 48 h, with the exception of *RPE65*, which underwent a marginal increase in expression at 48 h. These observations reveal striking stage-specific regulatory patterns associated with these RPE factors, although it remains to be detailed what mechanisms lead to the persistent increase in expression specific to E5 explants. One previous study using a similar explant system detailed how mesenchyme-derived TGF-β signals can continue to promote RPE differentiation in culture, and these observations could reflect the influence of a similar mechanism (Sakami et al., 2008). Nevertheless, the inability for E5 RPE to efficiently silence pigmentation machinery and shed identity characteristics in response to FGF2 is likely to have wide-reaching influences on neural competence and the underlying differentiation state.

Our present analysis further identified 1000s of regions of altered genomic accessibility across a narrow window of RPE development, and many of these regions have the potential to modify transcription factor activity in the RPE. The precise spatiotemporal control of extensive gene regulatory networks is necessary for the proper differentiation of both the RPE and neural retina lineages (Fuhrmann, 2010). The inner workings of these regulatory networks are incompletely resolved, although a recent study uncovered a remarkably complex RPE network that includes extensive cooperation of OTX2, MITF, and TEAD-family factors (Buono et al., 2021). Although we focus primarily on putative OTX2 binding sites, we identify numerous cis-regulatory elements that have the potential to exert stage-specific regenerative outcomes. Enhancers are known to be able to exhibit stage-specific activity and have the potential to directly affect regenerative outcome. For example, *Drosophila* imaginal discs robustly regenerate at the beginning of the third larval instar stage, but this ability is lost simultaneously with the silencing of injury-responsive enhancers during development (Harris et al., 2020). It is important to note that there are significant limitations to using chromatin accessibility as an indicator of transcription factor activity, such as was performed in the current study. The accessibility footprints associated with transcription factor binding events are ambiguous with regard to the *bona fide* identity of bound factors as well as the mechanisms of the associated regulatory interactions. Future work will further interrogate the identified cis-regulatory elements to detail their specific functions and how they may intersect with RPE neural competence.

Some of the accessibility changes detailed in the current study also have the potential to act as barriers to neural reprogramming or otherwise modify RPE behavior. Although we did not observe barriers to the transcriptional activation of neural retina factors within the measured time frames (6hPR *in vivo*), it is possible that regulatory barriers may further contribute to RPE identity at later stages of maturation. In this regard, mature murine RPE acquires epigenetic signatures that have the potential to act as barriers to gene activation necessary for reprogramming toward neural lineages, including through the placement of DNA methylation and repressive histone modifications within the promoters of key neural retina gene sets (Dvoriantchikova et al., 2019). We have demonstrated that overexpression of the DNA demethylation gene *TET3* is sufficient to induce E4 RPE reprogramming in the chicken in the absence of exogenous FGF2 (Luz-Madrigal et al., 2020), and it is probable that the intersection between DNA methylation and chromatin accessibility will be especially relevant to explaining neural competency at later stages of RPE differentiation. In the same study, it was demonstrated that DNA methylation is globally reset during embryonic RPE reprogramming, and it remains to be determined to what extent these alterations impact chromatin accessibility. Thus, it is likely that the epigenetic status of RPE cells contributes to neural competence beyond the mechanisms detailed in the present study, and the identification of specific epigenetic modifications that delineate E4 and E5 RPE will be fundamental to interpreting these results. Broadly, the observed decline in RPE neural competence parallels a recurring biological premise that organisms display a decrease in regenerative ability and cellular plasticity as they age (Yun, 2015). Ongoing work will build on these findings by detailing how specific regulatory features of RPE can be perturbed to expand neural plasticity and mammalian regenerative competency.

## Data Availability

The datasets presented in this study can be found in online repositories. The names of the repository/repositories and accession number(s) can be found here https://www.ncbi.nlm.nih.gov/geo/query/acc.cgi?acc=GSE197938.

## Supplementary Material

Supplementary Figure 1 High quality sequencing data was generated for RNA-seq analysis. **(A)** Average Phred quality score by base is summarized for the quality-trimmed RNA-seq reads. **(B)** The distribution of GC content in trimmed RNA-seq reads is shown, revealing a consistent distribution across all read sets. **(C)** Chart displays the alignment rate and number of total aligned reads for each RNA-seq sample using the chicken genome GRCg6a. **(D)** 3-dimensional principal component analysis plot summarizes the variation across samples using a normalized RNA-seq count matrix following variance stabilizing transformation. **(E)** Blind hierarchical clustering of RNAseq samples results in distinct clustering of samples by condition.

Supplementary Figure 2 Differential gene expression during RPE differentiation and reprogramming. **(A)** The RNA-seq normalized expression values for the top 5 genes previously reported as up regulated in E4 RPE 6hPR + FGF2 in an independently collected dataset (Tangeman et al., 2021) are displayed. **(B)** Similarly, the expression values of the top 5 down-regulated genes from the same study were plotted. Asterisk (*) denotes an adjusted *p*-value ≤ 0.05. **(C)** The scaled expression values for each gene in the 6 clusters defined by affinity propagation clustering shown in Figure 2 are displayed in line plots.

Supplementary Figure 3 Assessment of ATAC-seq data quality. **(A)** Chart displays the percent of duplicated reads, percent GC content, and total number of sequences associated with each raw read generated in the ATAC-seq analysis. **(B)** Figure displays the average Phred quality score of raw ATAC reads by read position for each read set. **(C)** Heatmaps display the normalized ATAC signal within 2 kilobases of all annotated transcription start sites (TSSs) in the chicken genome. **(D)** The fraction of reads in called peak regions (FRiP) score was calculated for each sample using the 89,823 consensus peaks called across all samples.

Supplementary Figure 4 Explant controls and developmental proliferation dynamics. **(A)** Two representative E4 and E5 explants are shown when cultured in the absence of FGF2 for 48 hours. No neural retina outgrowth is observed. **(B)** EdU and DAPI fluorescence staining was performed on sections from chick embryos collected at E3 (Hamburger Hamilton stage 21-22), E4 (Hamburger Hamilton stage 24), or E5 (Hamburger Hamilton stage 26). RPE and neuroepithelium (NE) are labeled below image. 50 μm scale bar applies to all panels. **(C)** Chart displays the proportion of EdU positive RPE cells observed at E3, E4 and E5. Diamond represents mean proportion; asterisk (*) denotes adjusted p value < 0.05. **(D)** GSEA of RNA-seq gene expression values obtained for intact E4 and E5 samples revealed a high enrichment of E2F targets in the E4 6hPR + FGF2 sample (normalized enrichment score = −3.00333).

Supplementary Figure 5 Changes in gene expression and accessibility reflect RPE maturation across the E4 to E5 window. GSEA of RNA-seq gene expression values obtained for intact **(A)** and 6hPR + FGF2 **(B)** samples reveals high enrichment of genes associated with fetal RPE in the E5 samples, with normalized enrichment scores of 2.5105283 and 2.2099848, respectively. **(C)** GSEA analysis of intact E4 and E5 RPE reveals a high normalized enrichment score (NES = 2.291688) for the gene set Integrin 1 Pathway, reflecting a high enrichment of genes encoding integrin receptors and extracellular matrix components in the E5 RPE. **(D)** Row-normalized heatmap displays RNA-seq gene expression values for integrin receptor genes and related extracellular matrix components. **(E)** Genome browser displays ATAC signal proximal to the promoters of 6 genes associated with RPE maturation.

Supplementary Figure 6 Accessibility and gene expression underscore AP1 factors as a hallmark of the RPE response to retinectomy and FGF2. **(A)** Gene expression values were binned into quintiles by gene expression (TPM) and the median accessibility signal proximal to transcript start sites (TSSs) were plotted for each bin. **(B)** DARs were annotated according to their genomic feature distribution. **(C)** Scatter plot displays the accessibility log fold change (LFC) of peak summit accessibility when comparing intact to 6hPR + FGF2 samples at E4 941 (x-axis) and E5 (y-axis). Only peak summits with adj. p-value ≤ 0.05 are shown. Overrepresented motifs in C were analyzed using the HOMER motif discover tool and top 15 motifs associated with accessibility changes at E4 **(D)** and E5 **(E)** are plotted. **(F)** RNA-seq normalized counts are plotted for JUN and FOS. Asterisk (*) indicates adjusted p. ≤ 0.05. y-axis is log-scaled.

Supplementary Figure 7 Specific transcription factor binding events are associated with differential gene expression. Differentially accessible peak summits were mapped to the nearest gene TSS, and genes with significant expression changes (adj. p < 0.05) were retained. Changes in accessibility and the accompanying change in gene expression were plotted. The regulatory logic linking differential accessibility to differential expression was inferred based on the quadrant in which each event is plotted (e.g., quadrant I in [A] contains genomic regions of increased E5 accessibility that are explicitly associated with increased E5 gene expression). HOMER motif analysis was used to identify overrepresented transcription factor binding motifs within each quadrant, which are displayed in tables for the developing **(A)** and 6hPR * FGF2 **(B)** conditions.

Supplementary Figure 8 Binned motif analysis reveals a diverse set of transcription factors associated with RPE maturation. The monaLisa motif enrichment tool was used with the JASPAR 2022 vertebrate motif database to plot motifs enriched in the E4 or E5 RPE. All motifs with -log(adj. p-value) > 3 in at least 1 peak bin are displayed for intact **(A)** and 6hPR + FGF2 **(B)** samples.

Supplementary Figure 9 Binned k-mer analysis overlaps with known transcription factor motifs. The monaLisa motif enrichment tool was used to detect enriched six nucleotide k-mers in the intact **(A)** or 6hPR + FGF2 **(B)** peak summits. Enriched k-mers with -log(adj. p-value) > 3 were matched to known motifs in the JASPAR 2022 database and similarity was plotted in the shown heatmaps.

## Abbreviations

DAR, differentially accessible region; DEG, differentially expressed gene; Dev., Developing; DIC, differential interference contrast; E3/4/5, embryonic day 3/4/5; GSEA, gene set enrichment analysis; 6hPR, 6 hours post-retinectomy; LFC, log fold change; PR, post-retinectomy; TPM, transcripts per million; TSS, transcription start sites.
