# Supplementary figures and images for: A Stage-Specific OTX2 Regulatory Network and Maturation-Associated Gene Programs Are Inherent Barriers to RPE Neural Competency
Source: Front Cell Dev Biol. 2022 Apr 19;10:875155. doi: 10.3389/fcell.2022.875155 (PMC9062105; doi:10.3389/fcell.2022.875155)

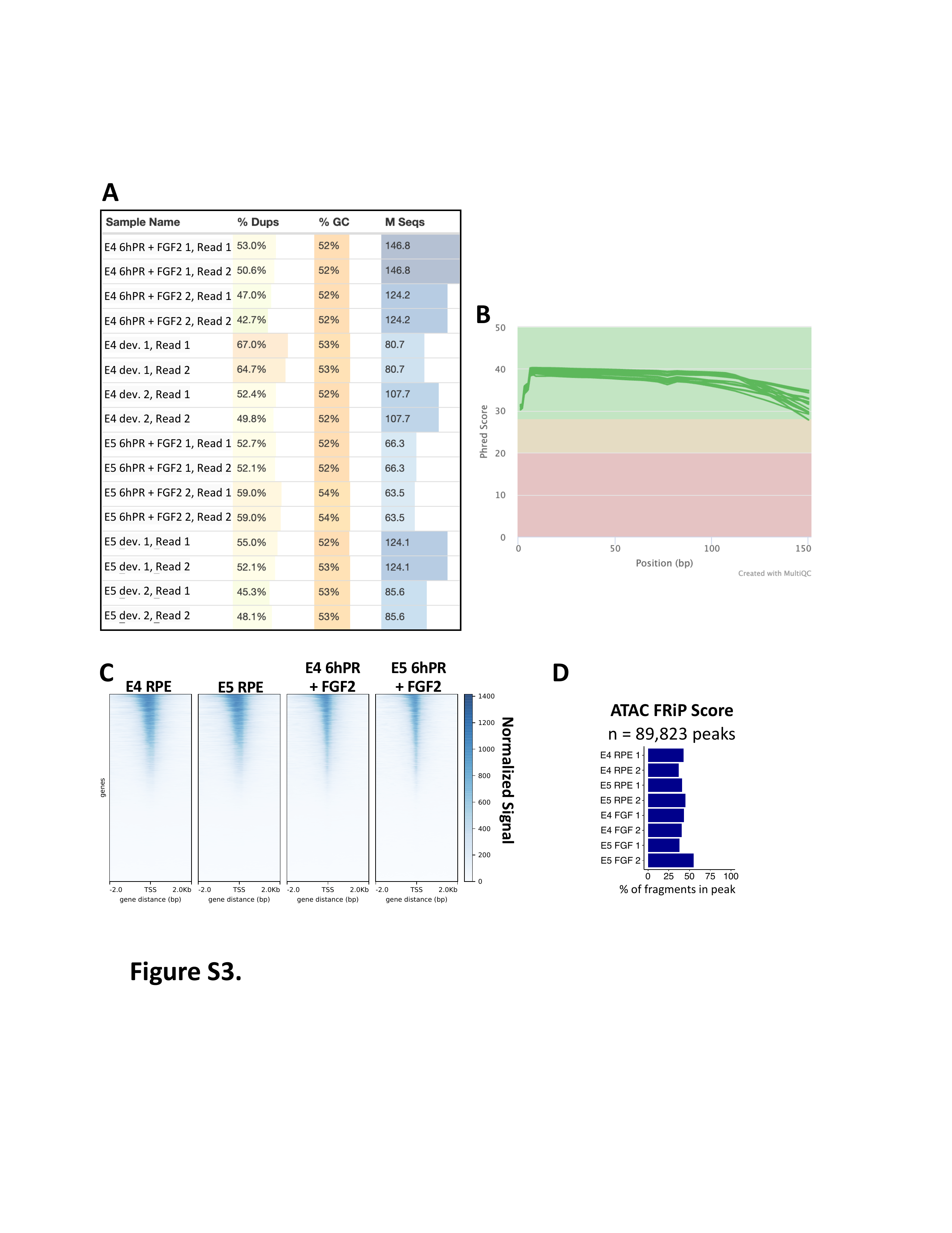

Supplement: Supplementary file 1 [file Image3.TIFF]

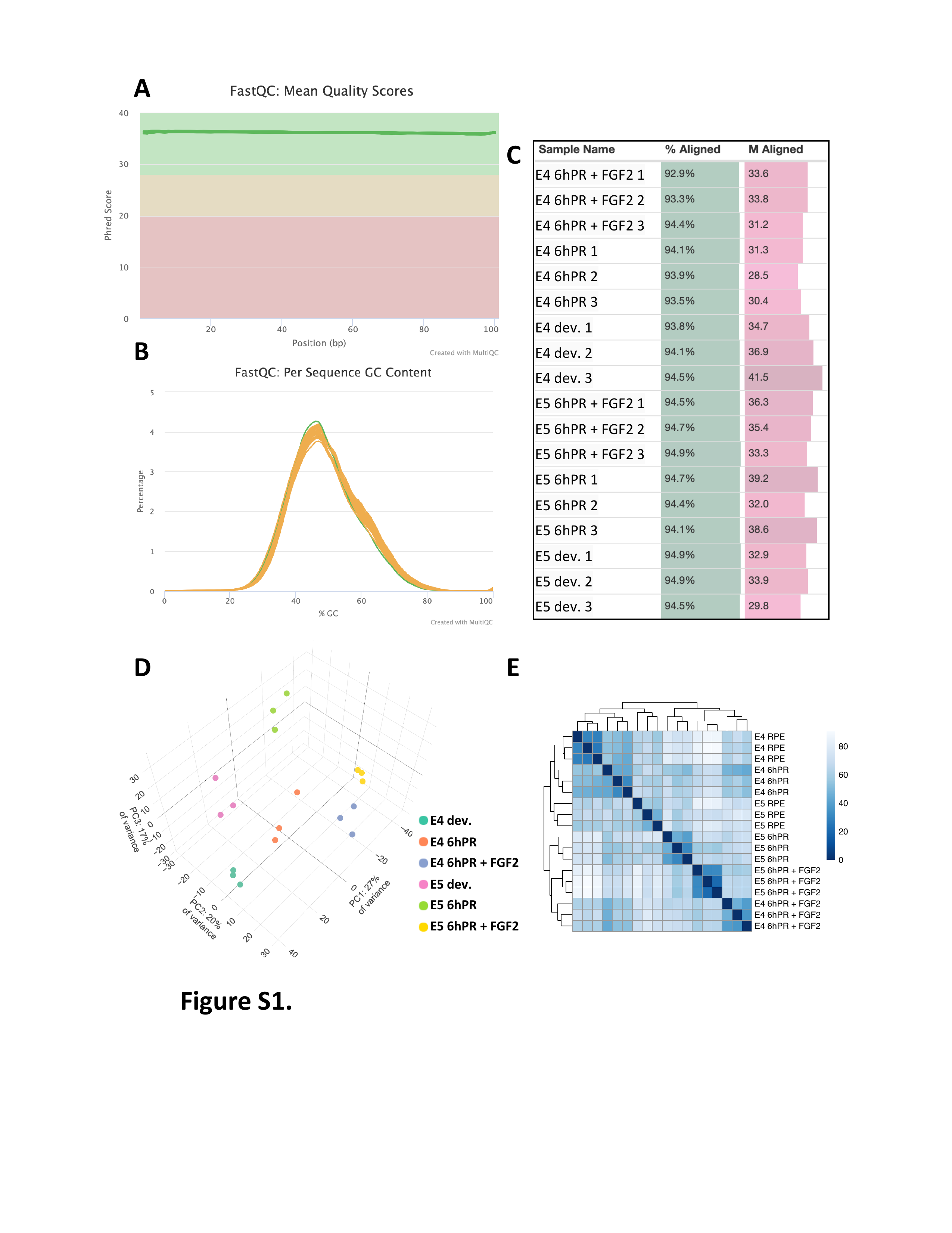

Supplement: Supplementary file 3 [file Image1.TIFF]

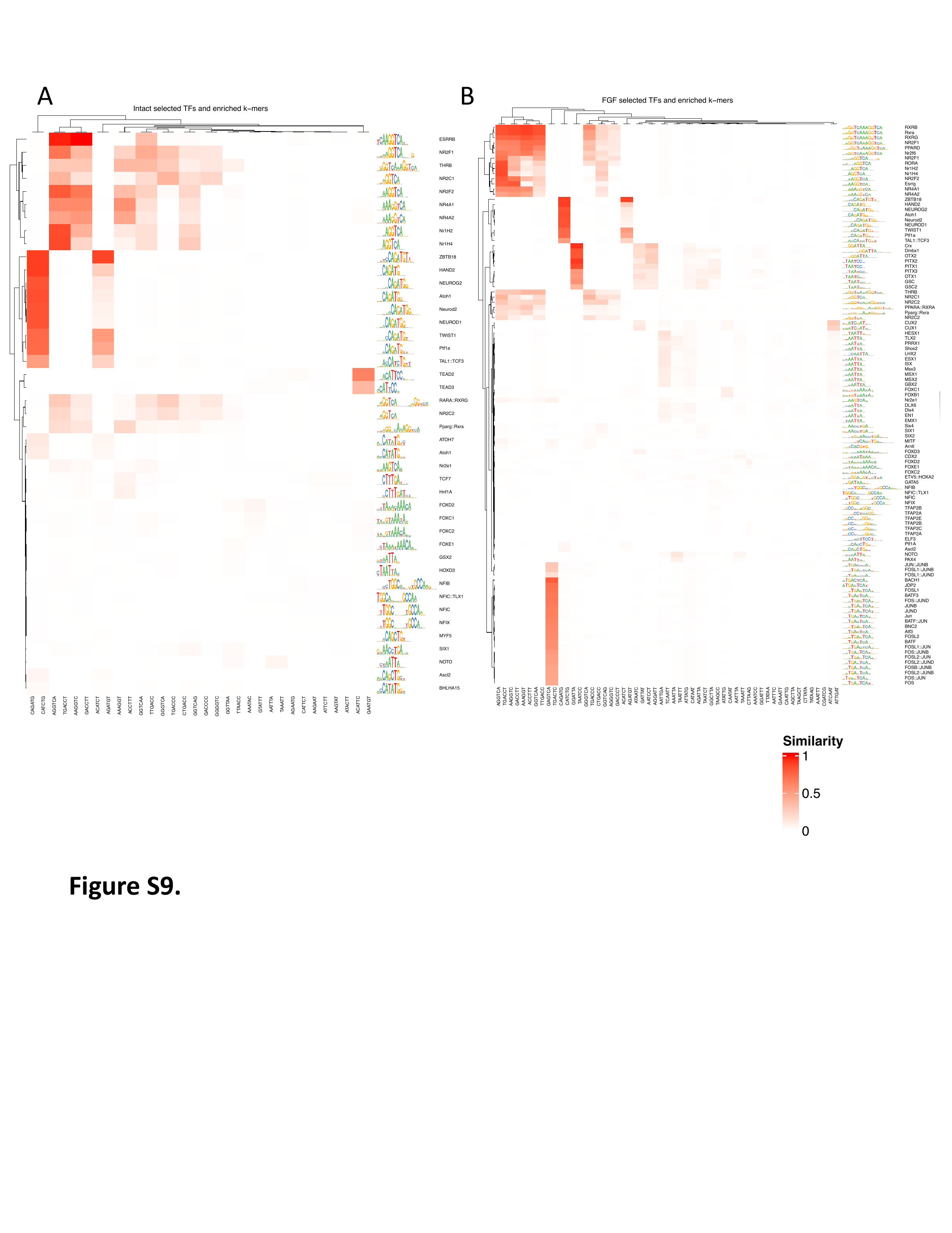

Supplement: Supplementary file 4 [file Image9.TIFF]

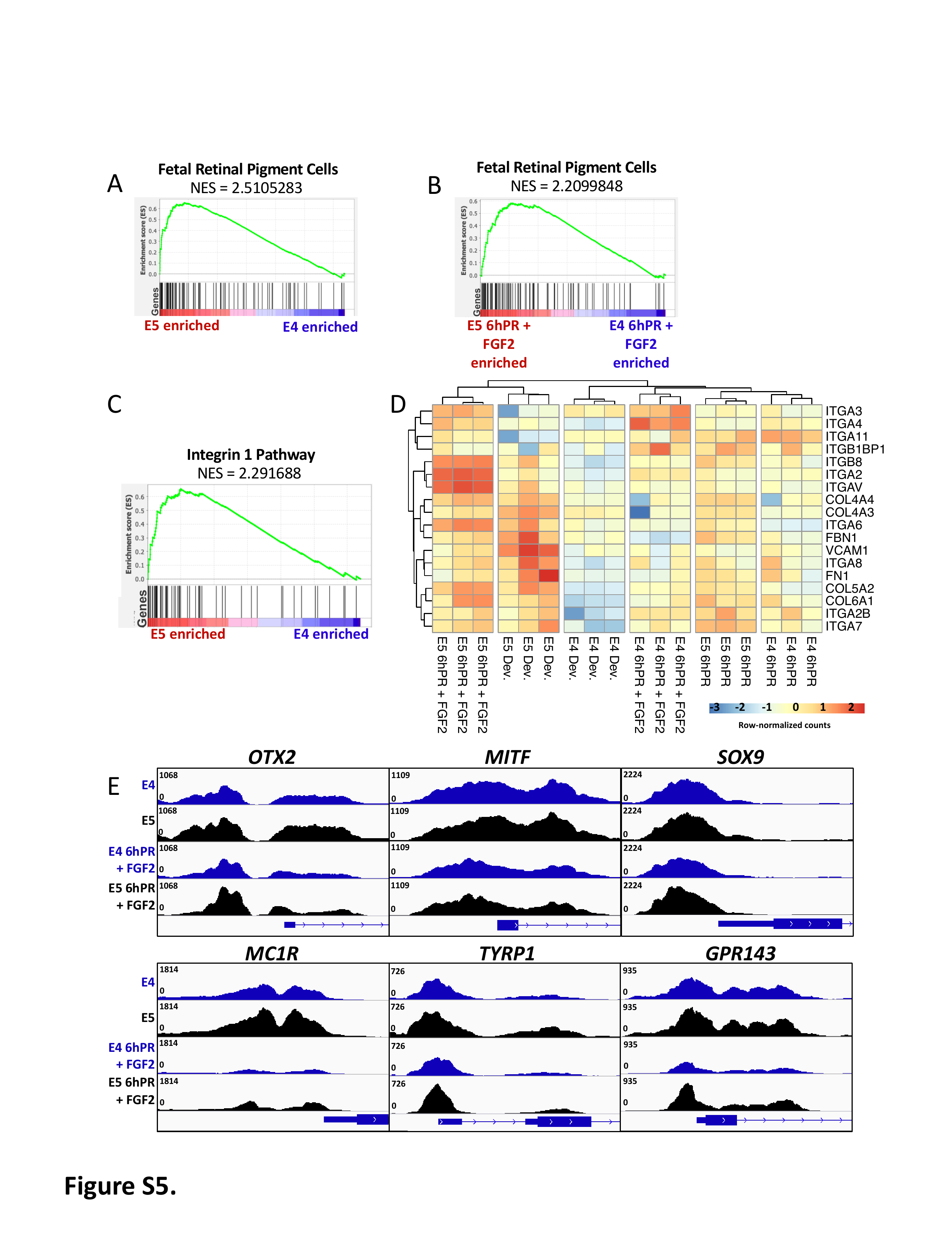

Supplement: Supplementary file 6 [file Image5.TIFF]

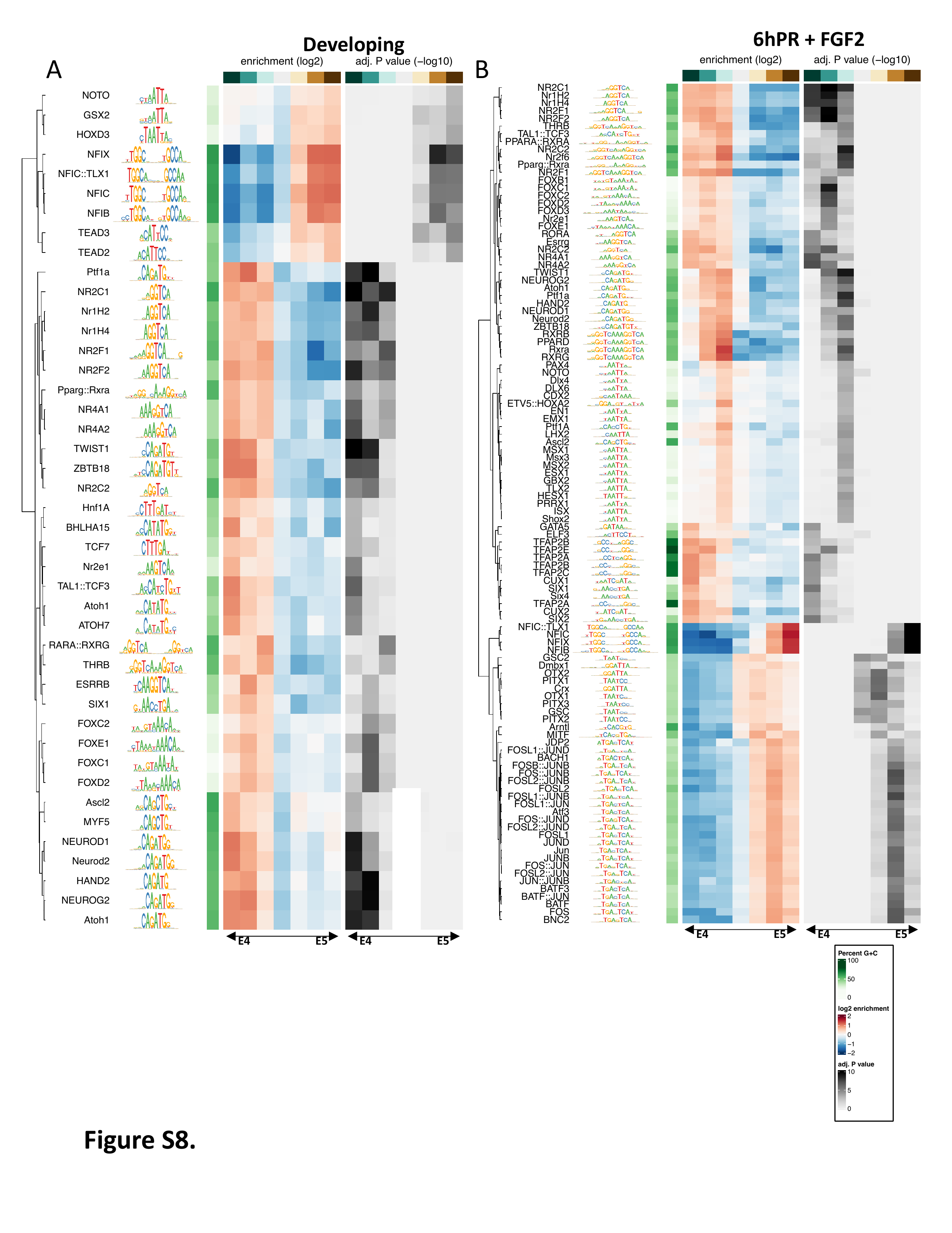

Supplement: Supplementary file 7 [file Image8.TIFF]

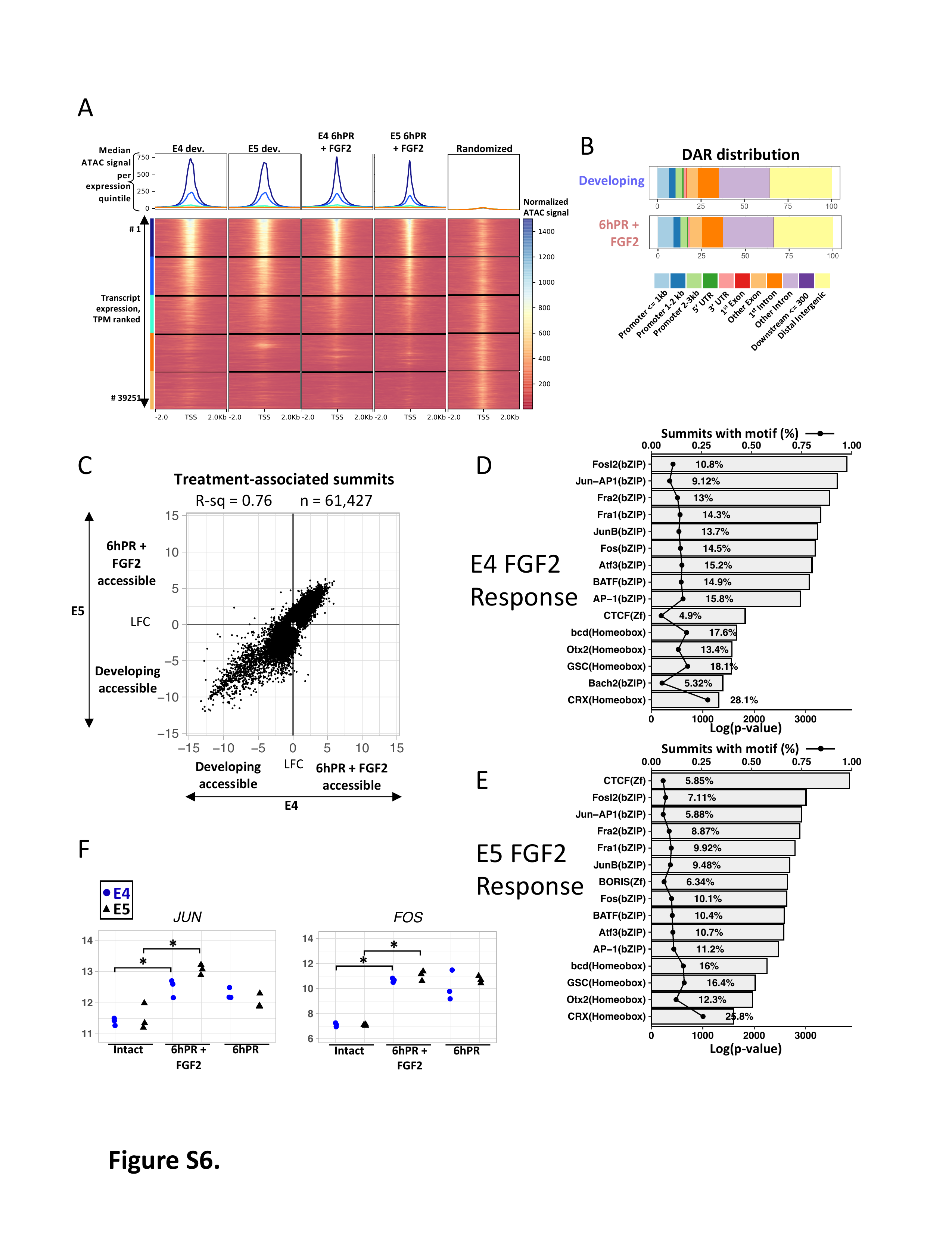

Supplement: Supplementary file 10 [file Image6.TIFF]

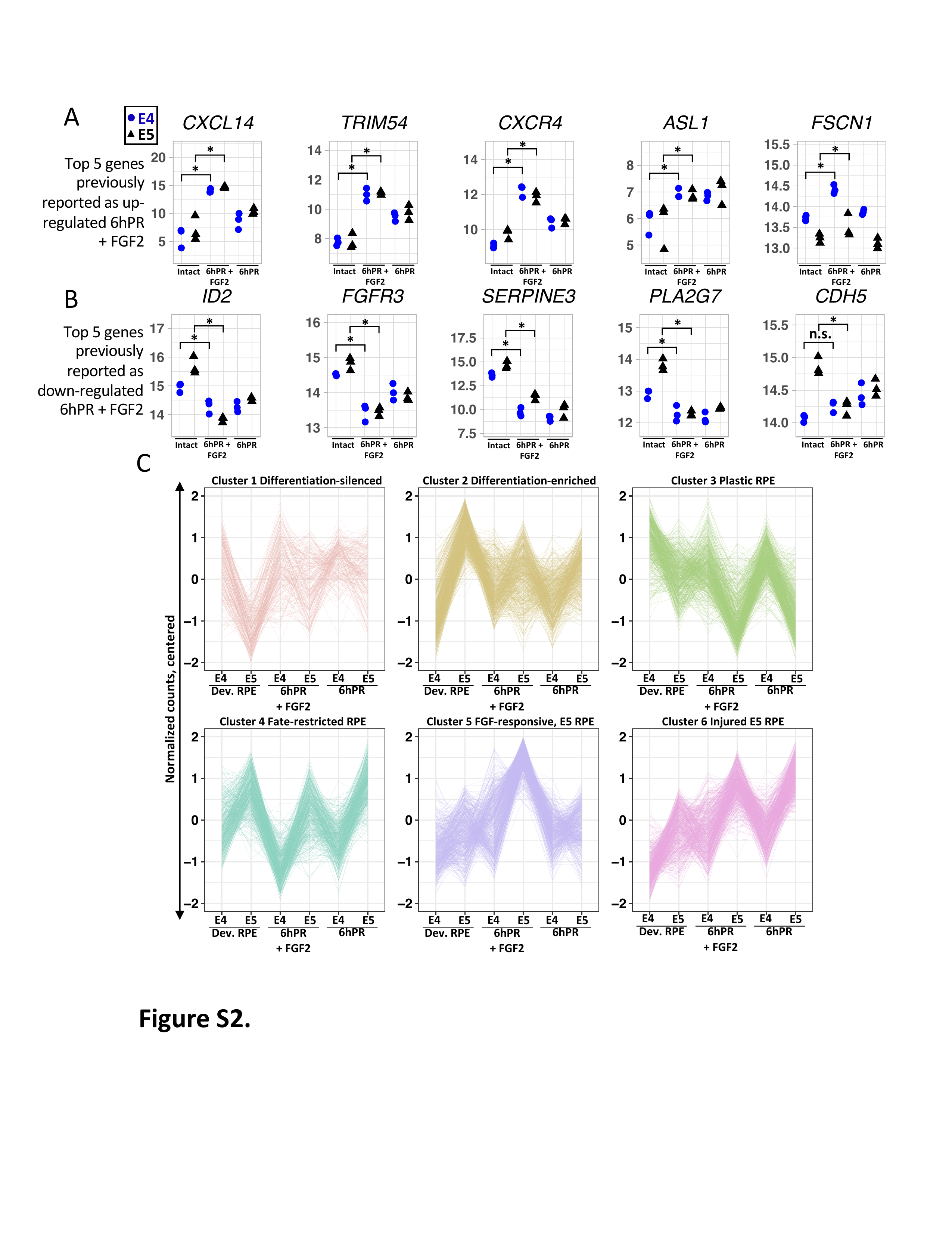

Supplement: Supplementary file 12 [file Image2.TIFF]

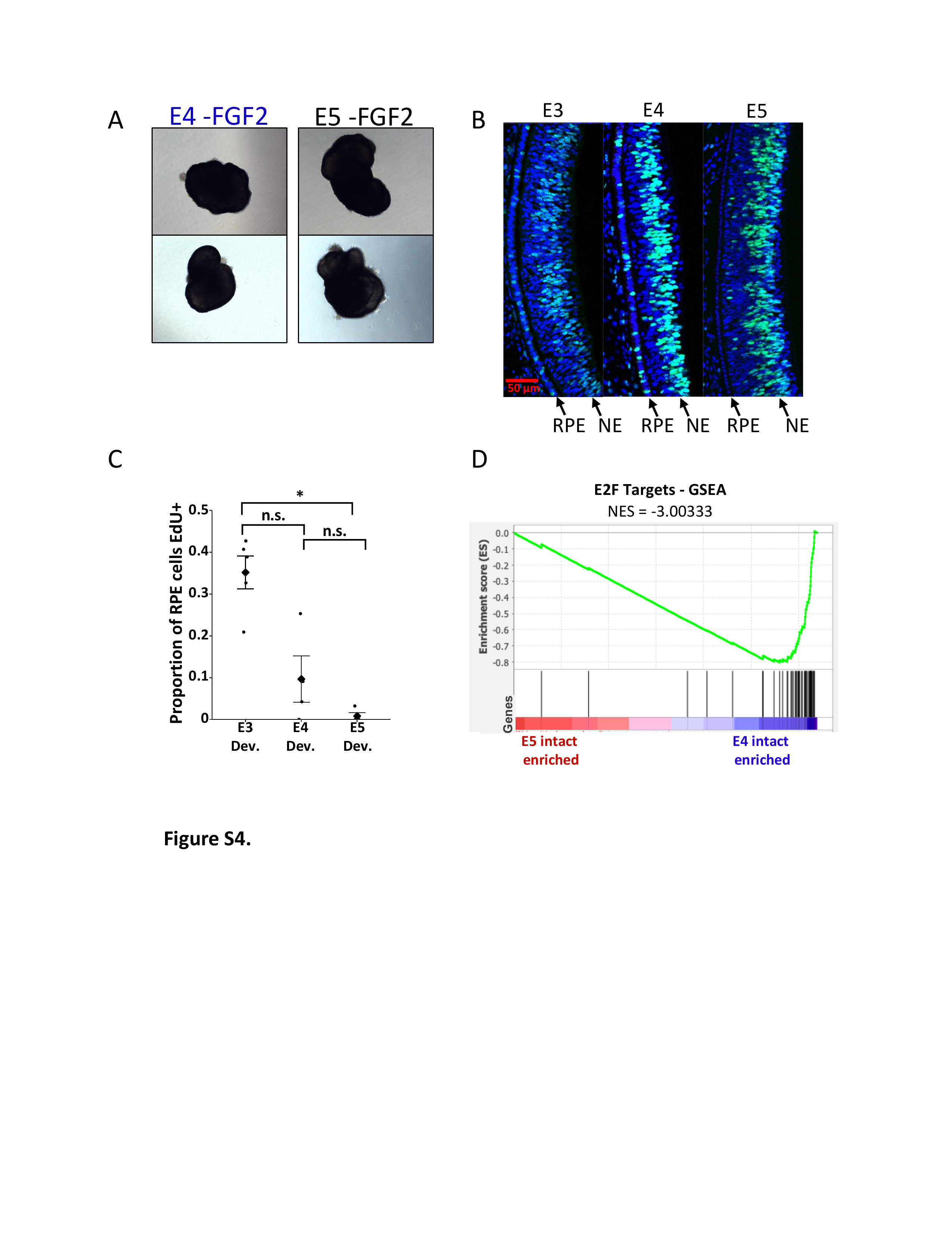

Supplement: Supplementary file 13 [file Image4.TIFF]

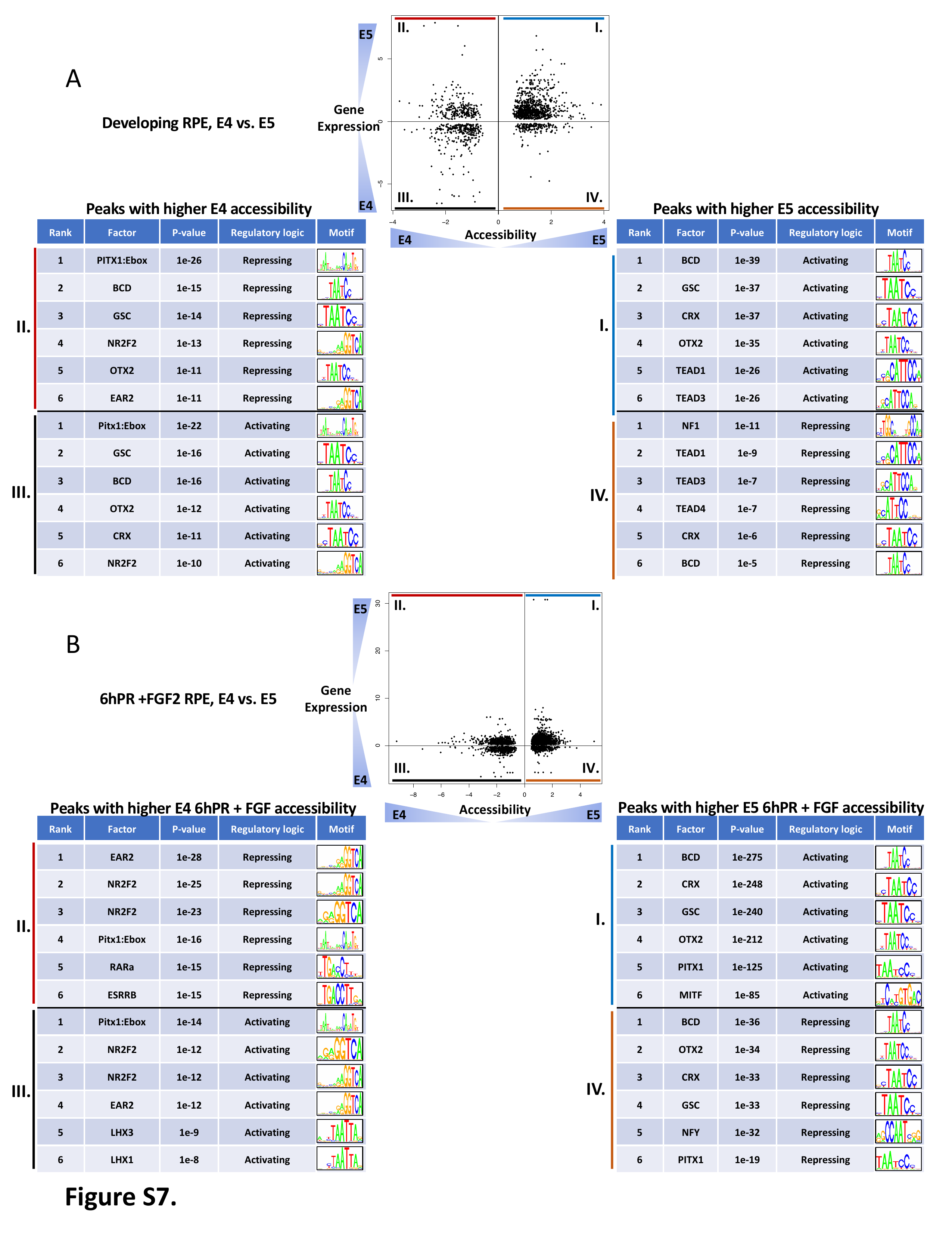

Supplement: Supplementary file 14 [file Image7.TIFF]
